# Correlation analysis of lung mucosa-colonizing bacteria with clinical features reveals metastasis-associated bacterial community structure in non-small cell lung cancer patients

**DOI:** 10.1186/s12931-023-02420-7

**Published:** 2023-05-11

**Authors:** Wenxue Wang, Xiao Liang, Hui Kong, Yun Yang, Yilan Xia, Qiongjiao Wang, Andong Xia, Jiawei Geng

**Affiliations:** 1 Department of Infectious Disease and Hepatic Disease, First People’s Hospital of Yunnan Province, Affiliated Hospital of Kunming University of Science and Technology, Jinbi Road #157, Kunming, Yunnan, 650032 China; 2 School of Medicine, Kunming University of Science and Technology, Kunming, Yunnan, 650500 China; 3 Faculty of Life Science and Technology, Kunming University of Science and Technology, Kunming, Yunnan, 650500 China

**Keywords:** NSCLC, Metastasis, bacteria, Community structure

## Abstract

**Background:**

Microbes colonizing lower airways can regulate the host immune profile and consequently participate in lung disease. Increasing evidence indicate that individual microbes promote lung cancer progression and are involved in metastasis incidence. To date, however, no study has revealed the community structure of lung bacteria in metastatic non-small cell lung cancer (NSCLC) patients.

**Methods:**

We prospectively enrolled 50 healthy subjects and 57 NSCLC patients. All healthy subjects and NSCLC patients underwent bronchoscope procedures for brush specimen collection. The 16 S ribosomal RNA gene was sequenced to characterize the community structure of lung mucosa-colonizing bacteria. The peripheral blood of NSCLC patients was also measured for leukocytes and cancer markers.

**Results:**

The lung bacteria of healthy subjects and NSCLC patients were divided into four communities. All community 2 members showed increased abundance in NSCLC patients compared with healthy subjects, and most community 2 members showed increased abundance in the metastatic NSCLC patients compared with the non-metastatic group. These bacteria were significantly and positively correlated with eosinophils, neutrophils and monocytes in the metastatic NSCLC group. In addition, the correlation between lung bacteria and cancer markers differed between the metastatic and non-metastatic NSCLC patients. Furthermore, bronchoalveolar lavage fluid from lung adenocarcinoma patients directly promoted NSCLC cell migration.

**Conclusions:**

The community structure of lung mucosa-colonizing bacteria was relatively stable, but changed from the healthy population to NSCLC patients, especially the metastatic group. This distinct community structure and specific correlation with immune cells and cancer markers could help to distinguish NSCLC patients with or without metastasis.

**Supplementary Information:**

The online version contains supplementary material available at 10.1186/s12931-023-02420-7.

## Background

Non-small cell lung cancer (NSCLC), which is primarily comprised of adenocarcinoma (AC) and squamous cell carcinoma (SCC), is the most common type of lung cancer (> 85%) and results in the most cancer-related deaths worldwide [1]. Although metastasis is the leading cause of poor prognosis in NSCLC patients [2], there are no effective strategies available for predicting early metastasis incidence in NSCLC. Notably, increasing evidence suggests that bacteria colonized around tumors participate in NSCLC metastasis both directly and indirectly [3, 4], as illustrated by the tight connection between bacterial colonization and tumor-infiltrating immune cells [5]. To date, however, few studies have explored the correlation networks between bacteria colonized around tumors and host immune cells with metastasis incidence in NSCLC patients or evaluated the clinical potential of these correlations in the diagnosis of metastasis.

Bioinformatics is a powerful tool that can be applied to discover the correlation between bacteria and NSCLC progression. Using bioinformatics analysis, a recent study found that lung bacteria, such as *Veillonella parvula*, can modulate host immune profiles and affect NSCLC progression and prognosis [6]. Because tumor-infiltrating immune cells are delivered via peripheral blood [7], bacteria colonizing around tumor could affect the composition of immune cells in serum and impact following NSCLC progression. Thus, systematic analysis of the correlation between immune cell profiles in peripheral blood and community structure of lung mucosa-colonizing bacteria is more valuable in predicting NSCLC metastasis incidence.

In addition, carcinoembryonic antigen (CEA), a typical marker of lung cancer, has application potential not only in the diagnosis of carcinogenesis but also in predicting early metastasis [8]. Combined analysis of CEA and other clinical features could significantly increase the reliability of metastasis prediction [9]. In this study, NSCLC patients and healthy individuals were recruited to characterize lung mucosa-colonizing bacterial communities. We compared the correlation of lung mucosa-colonizing bacteria with immune cells and typical cancer markers in NSCLC patients with or without metastasis. We also investigated the effects of bronchoalveolar lavage (BAL) fluid obtained from NSCLC patients with metastasis on the migration of cancer cells.

## Methods

### Patients and study design

Healthy participants and NSCLC patients were recruited at the First People’s Hospital of Yunnan Province, China, between January 2015 to October 2017. Because smoking and occupational exposure to dust are the main risk factors of lung cancer [10], each participant provided information on age, occupation, smoking history (daily cigarettes and smoking period), and family history of lung cancer. The healthy population had no history of pulmonary disease or ongoing serious medical illnesses and showed normal spirometry. Each patient was diagnosed with NSCLC but reported no chemotherapeutic experience or antibiotic use (within three months). All participants provided signed informed consent and finished the questionnaire regarding their age, sex, occupation, and antibiotic use, with family assistance if necessary.

*Diagnosis of NSCLC* A diagnosis of NSCLC was made after multidisciplinary team discussion, according to the latest clinical practice guidelines [11, 12]. The chief complaints of outpatients were first investigated, which mainly included cough, hemoptysis, asthma, fever, and chest pain. Based on the assessment of lung cancer risk factors, outpatients with high risk due to occupational exposure, smoking, and family history of lung cancer received an immune cell and cancer marker test via peripheral venous blood. Chest computed tomography (CT), which is commonly used in the diagnosis of lung cancer, can also effectively detect early peripheral lung cancer and clarify lesion location and range [13]. Therefore, patients also underwent enhanced chest CT when the immune cell and cancer marker test suggested further screening of lung cancer. Patients were also advised to quit smoking and introduce a healthier diet and lifestyle. Bronchoscopies were performed to survey bronchial lesions and collect brush, lavage fluid, and biopsy samples, which were used for immunohistochemical and histopathological analysis. For SCC diagnosis, tissues showed clear morphological patterns of unequivocal keratinization and well-formed classical bridges as well as P40 expression via histopathological and immunohistochemical analysis, respectively. For AC diagnosis, tissues showed clear morphological patterns (i.e., acinar, papillary, lepidic, and micropapillary) via histopathological analysis; in addition, thyroid transcription factor-1 (TTF-1) was used to distinguish primary versus metastatic AC in immunohistochemical analysis [1].

*Diagnosis of distant metastasis* Lymph node (mediastina, hilar, and cervical) metastasis was confirmed by both ultrasonography-directed biopsy and CT strategies. The NSCLC patients with hepatomegaly and liver pain symptoms accompanied by low appetite, nausea and weight loss, and elevated aspartate serum levels of aminotransferase or bilirubin [14], received both CT and nuclear magnetic resonance imaging (NMRI) tests to confirm liver metastasis. NSCLC patients with chronic pain and tenderness of the ribs, spine, pelvis, and long bone [15], received NMRI and radionuclide bone imaging (if necessary) to confirm bone metastasis. NSCLC patients with typical brain disease symptoms, including headache, vomiting, dizziness, diplopia, ataxia, hemiplegia, and epilepsy [14], received NMRI (enhanced if necessary) to confirm intracranial metastasis.

*Eligibility Criteria* Inclusion criteria were: histologically or cytologically proven NSCLC. All tumor sites (local, regional, and distant) had to be amenable for radical treatment according to the multidisciplinary team. There were no size limitations to the primary tumor or its metastases. Exclusion criteria were: Most NSCLC patients were excluded due to antibiotic use within the last three months. NSCLC patients with histories of tuberculosis, chronic obstructive pulmonary disease, and pneumoconiosis were also excluded. In addition, patients diagnosed with small cell lung cancer, large cell neuroendocrine carcinoma, or non-primary lung tumors were also excluded.

*Sample collection* Briefly, NSCLC patients and healthy participants were first given nebulized oropharyngeal anesthesia, then bronchoscopy was applied to localize lesion sites or designative lobes. A specimen brush attached to bronchoscopy was extended for sampling mucosa and bronchoalveolar lavage (BAL) fluid. For NSCLC patients, mucosa-brushing samples were collected from tumor niduses. For healthy participants, mucosa-brushing samples were collected from five lobes in turn. All mucosa samples, which were collected from January 2015 to October 2017, were immediately placed on ice and frozen within 1 h at − 80 °C. Bronchoalveolar lavage samples were used for transwell migration assays within 4 h of collection from NSCLC patients.

### Bacterial DNA isolation and 16 S rRNA gene sequencing

Microbial community genomic DNA was extracted from brush samples of lung mucosa using a QIAamp DNA Mini Kit (Qiagen, Germany) according to the manufacturer’s instructions. The DNA extract was checked on 1% agarose gel, and DNA concentration and purity were determined with a NanoDrop 2000 UV-vis spectrophotometer (Thermo Scientific, Wilmington, USA). The hypervariable region V3-V4 of the bacterial 16S rRNA gene was amplified with primer pairs 338F (5’-ACTCCTACGGGAGGCAGCAG-3’) and 806R (5’-GGACTACHVGGGTWTCTAAT-3’) using an ABI GeneAmp® 9700 PCR thermocycler (ABI, CA, USA). PCR amplification of the 16 S rRNA gene was performed as follows: initial denaturation at 95 °C for 3 min, followed by 27 cycles of denaturing at 95 °C for 30 s, annealing at 55 °C for 30 s, extension at 72 °C for 45 s, single extension at 72 °C for 10 min, and end at 4 °C. The PCR mixture contained 5 × *TransStart* FastPfu buffer 4 µL, 2.5 mM dNTPs 2 µL, forward primer (5 µM) 0.8 µL, reverse primer (5 µM) 0.8 µL, *TransStart* FastPfu DNA Polymerase 0.4 µL, template DNA 10 ng, and finally ddH_2_O up to 20 µL. PCR was performed in triplicate. The PCR products were extracted from 2% agarose gel and purified using an AxyPrep DNA Gel Extraction Kit (Axygen Biosciences, Union City, CA, USA) according to the manufacturer’s instructions and quantified using a Quantus™ Fluorometer (Promega, USA).

Purified amplicons were pooled in equimolar concentrations and paired-end sequenced on an Illumina MiSeq PE300 platform/NovaSeq PE250 platform (Illumina, San Diego, USA) using standard protocols.

### Processing of sequencing data

The raw 16 S rRNA gene sequencing reads were demultiplexed, quality-filtered by fastp v0.20.0 [16], and merged by FLASH v1.2.7 [17] with the following criteria: (i) 300-bp reads were truncated at any site receiving an average quality score of < 20 over a 50-bp sliding window, and the truncated reads shorter than 50 bp were discarded, reads containing ambiguous characters were also discarded; (ii) only overlapping sequences longer than 10 bp were assembled according to their overlapped sequence. The maximum mismatch ratio of overlap region is 0.2. Reads that could not be assembled were discarded; (iii) Samples were distinguished according to the barcode and primers, and the sequence direction was adjusted, exact barcode matching, 2 nucleotide mismatch in primer matching.

Operational taxonomic units (OTUs) with 97% similarity cutoff [18, 19] were clustered using UPARSE v7.1, and chimeric sequences were identified and removed. The taxonomy of each OTU representative sequence was analyzed by RDP Classifier v2.2 [20] against the 16 S rRNA database (Silva v132) using a confidence threshold of 0.7. To assess background contamination, we sequenced three samples of pure water as a negative control with the same procedures, including DNA extraction, PCR amplification, cDNA library structure, and final sequencing. According to the sequencing results of the negative control, the same DNA sequence count detected in the negative control was extracted from all healthy and NSCLC samples. The sequencing data were submitted to SRA under BioProject accession number: PRJNA667552 (https://www.ncbi.nlm.nih.gov/bioproject/ PRJNA667552).

### Bioinformatics analysis

*Defining community structure of lung mucosa-colonizing bacteria* Variation in microbiota composition at the genus level was assessed. The abundances of the 50 highest-ranked bacterial genera were used to generate a bacterial network. The correlation values between different bacterial genera were identified using Spearman rank correlation coefficients [21], where absolute bacterial abundances were bootstrapped 999 times to generate correlation *P* values. Bacterial networks were then generated from selected correlations (|r|>0.5) and *P* values (*P* < 0.05) using Cytoscape v3.4.0. Firstly, bacteria, that formed a close and dense net, were classified as the members of one unique community. Secondly, the bacteria belonging to one unique community were required to positively correlate with one or more bacteria of same community, the bacteria correlated negatively were excluded from the defined community. Thirdly, the unique community independently formed a correlative net within inner members, and did not correlate or only negatively correlate with other communities. Based on these three principles, the lung mucosa-colonizing bacteria were divided into four communities, including community 1 (core members: *Saccharimonadales*, *Sphingomonas*, *Bradyrhizobium*), community 2 (core members: *Neisseria*, *Alloprevotella*, *Prevotella*, *Peptostreptoccus*, *Haemophilus*, *Granulicatella*), community 3 (core members: *Burkholder-Caballeronia-Paraburkholderia*, *Acinetobacter*, *Rhodococus*) and community 4 (core members: *Prevotella*, *Lactobacillus*, *Megamonas*).

*Correlation heatmap/network of bacteria-immune cells or bacteria-cancer markers* Using the R package [22], correlation heatmaps/networks were constructed based on Pearson correlation coefficients between bacterial relative abundances at the genus level and either immune cell counts or cancer marker assay data. For putative biologically relevant associations, only taxa detected in at least two samples were included. A filter was applied to select only associations with Pearson coefficient |r|>0.5 and *P* < 0.05.

All bioinformatics analysis in this study were performed on the online platform of Majorbio Cloud Platform (www.majorbio.com)[23].

### Immune cell and cancer marker measurement

The cell densities of five immune cell types (i.e., leukocytes, neutrophils, eosinophils, lymphocytes, and basophils) in peripheral blood were measured using standard instrumental (XN-1000, Sysmex, Japan) procedures. Measurements of four cancer markers (SCCA, CEA, NSE, and CYFRA21-1) in peripheral blood were processed using test kits (Autobio, China) and instruments (Autolumo A2000, Autobio, China) according to the manufacturers’ manuals. CA125 concentration was individually tested with an Elecsys CA 125 II (Roche, Germany) and supporting instruments (Cobas e 602, Roche, Germany).

### Transwell migration assay

The H1299 cells were kindly provided by the Cell Bank/Stem Cell Bank, Chinese Academy of Sciences. Dulbecco’s Modified Eagle Medium (DMEM, 600 µL) containing 20% fetal bovine serum (FBS) was placed in the lower compartment of a 24-well plate. A cell suspension (1 × 10^5^) of NCI-H1299 in 200 µL of DMEM without FBS was placed in the upper compartment of the transwell inserts, followed by 50 µL of freshly collected bronchoalveolar lavage, as well as kanamycin (5 µg/L) and vancomycin (2.5 µg/L) according to grouping arrangement. The upper and lower compartments were separated by a 6.5-mm polycarbonate filter with an 8-µm pore diameter (CLS3422, Corning, Sigma, USA). After 48 h, the cells that had not migrated to the lower chamber were scraped off with a cotton swab. The filters were fixed with 4% paraformaldehyde solution for 30 min and the cells were then stained with 0.1% crystal violet staining solution for 20 min. The stained transwell membranes were cut, mounted on microscope slides, and analyzed under a microscope to determine the number of migrated cells. Four nonoverlapping fields were analyzed using Image J software to determine average number of migrated cells.

### Statistical analysis

GraphPad Prism 9.0.0 (San Diego, CA, USA) and the R stats package were used to analyze all data [24]. Wilcoxon rank sum tests with false discovery rate (FDR) correction (bootstrap, CI = 0.95) for multiple comparisons were conducted to identify differentially abundant bacterial taxa. Differences in bacterial relative abundance among the healthy population, NSCLC patients with and without metastasis, and SCC and AC patients, were tested using multiple unpaired *t*-tests, which were adjusted using the two-stage step-up method (Benjamini, Krieger, and Yekutieli). Transwell migration data were analyzed using two-way analysis of variance (ANOVA) with *post hoc* Tukey tests. Pearson rank-order correlation was used to evaluate associations between genus-level bacterial relative abundances and immune cell counts and cancer marker concentrations. Two-tailed student’s *t-*tests were used to compare immune cell counts, cancer marker concentrations, and degree and clustering of bacterial network. In all analyses, *P* < 0.05 was considered statistically significant.

## Results

### Subjects, Sampling, and sequencing

Fifty-seven patients diagnosed with NSCLC and 50 healthy subjects were enrolled in this study. All provided signed informed consent for bronchoscopy and other clinical tests (Table 1). The NSCLC patients included 33 SCC patients (all males, aged 49 to 80 years) and 24 AC patients (22 males and two females, aged 33 to 64 years). The enrolled NSCLC patients and healthy subjects were inquired face-to-face (not questionnaire) regarding their antibiotic use in the most recent three months, and those providing a positive answer were excluded from the study. The NSCLC patients included 40 farmers, six factory workers, six miners, and five subjects with other occupations. Most NSCLC patients (52 out of 57) had a history of smoking, with 46 patients smoking > 3600 cigarettes one year. The chief complaint among these patients included cough (24 individuals), hemoptysis (16 individuals), asthma (two individuals), fever (two individuals), and chest pain (four individuals). Seventeen NSCLC patients were diagnosed with intrapulmonary, thoracic and/or cervical lymph node metastasis, and four AC patients had multiple metastatic sites, including liver, bone, and intracranial. Eighteen patients died of NSCLC within 6 months of their diagnosis, and only nine patients survived over 24 months. All 107 lung mucosa brush specimens from 50 healthy subjects and 57 NSCLC patients underwent sequencing of the V3-V4 region of the 16 S rRNA gene, and detail amplicon information was shown in Table S1.

**Table 1:** Basic characteristics of NSCLC patients and healthy volunteers

Index	SCC patients		AC patients		Healthy volunteers (n = 50)
	No met (n = 20)	Met (n = 13)	No met (n = 6)	Met (n = 18)	
Age (years)	63 ± 14	60 ± 12	61 ± 4	49 ± 16	53 ± 21
Gender (M:F)	20:0	13:0	6:0	16:2	25:25
Antibiotic use (within 3 months)	No	No	No	No	No
*Occupation*					
Farmer	18	8	6	8	23
Factory worker	2	2	0	2	0
Miner	0	2	0	4	0
Other	0	1	0	4	27
*Smoking (daily)*					
0 cigarettes	3	0	0	2	50
1 ~ 10 cigarettes	2	0	0	4	0
10 ~ 20 cigarettes	4	2	6	8	0
20 ~ 30 cigarettes	9	8	0	4	0
≥ 30 cigarettes	2	3	0	0	0
*Chief complaint*					
Cough	6	6	2	10	
Hemoptysis	8	4	2	2	
Asthma	2	0	0	0	
Fever	0	0	0	2	
Chest pain	2	0	2	0	
Other	2	3	0	2	
*Metastasis*					
Lung inner	-	0	-	2	
Liver	-	2	-	2	
Bone	-	2	-	2	
Lymph nodes (hilar, mediastinal, and cervical)	-	9	-	8	
Multiple sites (liver, bone, and intracranial)	-	0	-	4	
*Survival (months)*					
≤ 6	8	4	2	4	
6 ~ 12	0	3	2	10	
12 ~ 24	5	6	0	4	
≥ 24	7	0	2	0	

NSCLC, non-small cell lung adenocarcinoma; SCC, lung squamous cell carcinoma; AC, lung adenocarcinoma; M, male; F, female; Met, metastasis; Thorax includes pleura, mediastinal, and hilar lymph nodes

### Alpha and beta diversity

Increasing evidences indicate that lung commensal microbiota exhibits a different community after carcinogenesis incidence and participates subsequent NSCLC progress [6, 25]. Consistent with these studies, lung mucosa-colonizing bacteria composition of NSCLC patients was significantly different with which of healthy population in our study. Beside decreased Shannon indexes (*P* = 0.00447) (Figure S1A), principal coordinate analysis (PCoA) of lung mucosa-colonizing bacteria was also different (*P* = 0.03) between healthy population and NSCLC patients (Figure S1B).

### Bacterial community structure

**Fig. 1:**
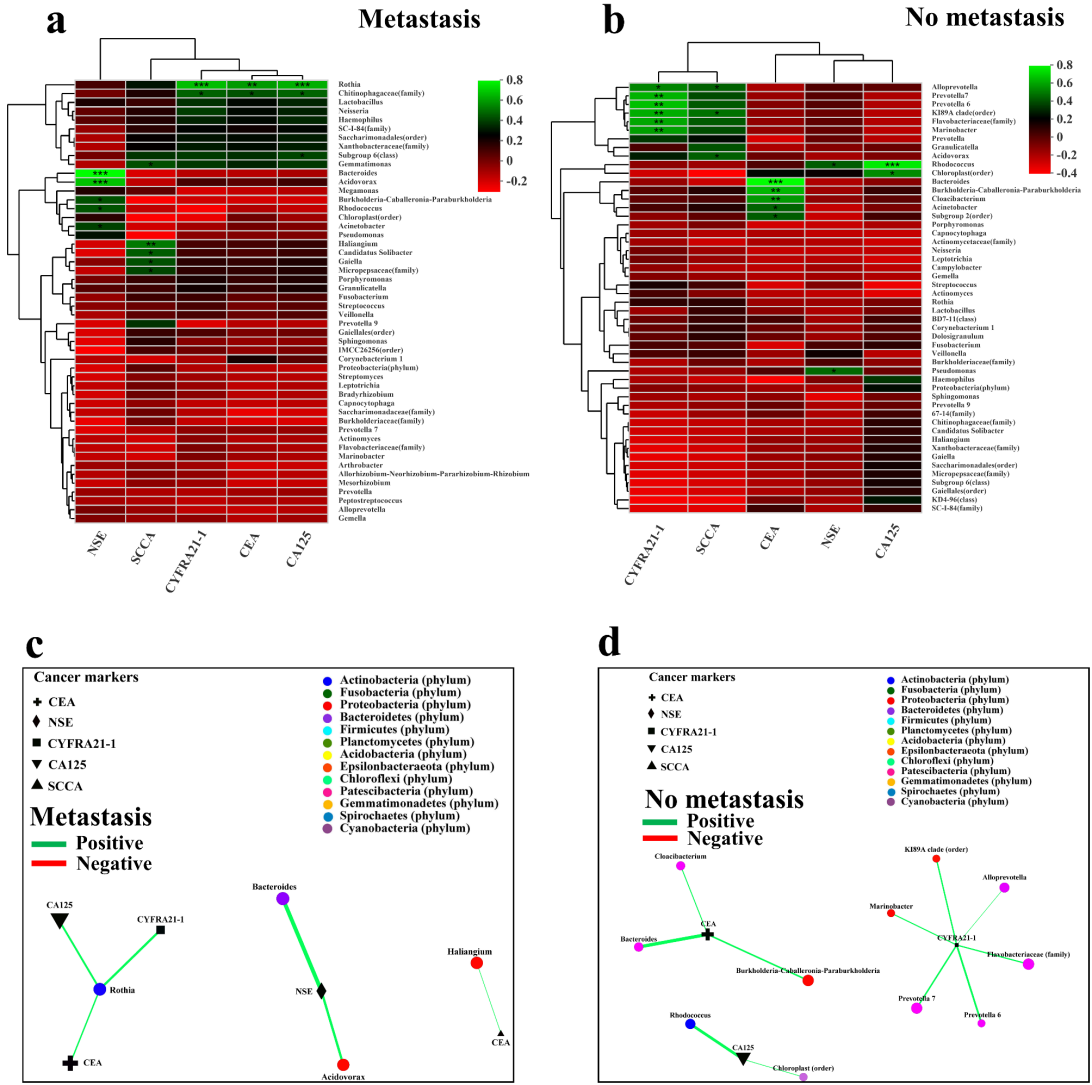
**Community structure of lung mucosa-colonizing bacteria and correlation between community members and immune cells in NSCLC patients.** (**A**) Correlation network of mucosa-colonizing bacteria in NSCLC patients (n = 57). Correlation network was constructed based on Spearman rank correlation coefficients (∣Spearman Coef∣ ≥ 0.5, *P* < 0.05). Dotted lines encircle different bacterial communities containing relatively stable inner members that are positively correlated with each other. Node diameter is positively related to bacterial abundance. Node colors indicate different bacterial phyla. Lines connecting different nodes indicate positive (green) or negative (red) correlation between bacteria, and line diameter is positively related to correlation value. Abundance comparison of community 1 **(B)**, 2 **(C)**, 3 **(D)**, and 4 **(E)** members between healthy populations (n = 50) and NSCLC patients (n = 57). Y axis represents relative abundance percentage (mean proportion) of each bacterial genus in healthy populations and NSCLC patients. * 0.01 < *P* ≤ 0.05, ** 0.001 < *P* ≤ 0.01, confidence interval (CI) = 0.95. Correlation network and heatmap contain top 50 abundant bacteria. All bacteria were named to genus level unless noted otherwise in brackets

Bacterial network analysis can help distinguish different types of lung disease, such as neutrophilic asthma and eosinophilic asthma [26]. We previously applied bacterial network analysis to successfully investigate the spatial heterogeneity of human intestine mucosa-colonizing bacteria [27] and their possible contribution model on local tissue neoplastic progress [28]. Here, we applied similar method to analyze the lung mucosa-colonizing bacteria of healthy subjects and found they were divided into four basic communities (i.e., communities 1, 2, 3, and 4). Because these communities not only had stable community members, but also these members within each community formed a positively and densely correlated network, and each community had no positive correlation with other communities. Community 1 was a core community composed predominantly of five phyla, including *Proteobacteria* (genera *Sphingomonas*, *Bradyrhizobium*, *Haliangium*), *Actinobacteria* (genera *Streptomyces*, *Gaiella*, *Arthrobacter*), *Acidobacteria* (genus *Candidatus Solibacter*), and *Patescibacteri*a (order *Saccharimonadales*). Community 2 was a core community comprised mainly of four phyla, including *Bacteroidetes* (genera *Prevotella 7*, *Alloprevotella*, *Porphyromonas*), Proteobacteria (genera *Haemophilus*, *Neisseria*), *Firmicutes* (genera *Streptococcus*, *Veillonella*), and *Fusobacteria* (genera *Fusobacterium*, *Actinomyces*). Community 3 was mainly comprised of two phyla including *Proteobacteria* (genera *Acinetobacter*, *Acidovorax*) and *Actinobacteria* (genus *Rhodococcus*), while community 4 was mainly comprised of two phyla including *Firmicutes* (genera *Lactobacillus*, *Megamonas*, *Faecalibacterium*) and *Bacteroidetes* (genera *Bacteroides*, *Prevotella 9*) (Figure S2, 4 C left pane). Interestingly, same bacterial community membership was also shown in NSCLC patients, lung mucosa-colonizing bacteria of which were divided into four communities and each community had same bacterial member of healthy subjects (Figs. 1A and 4 C middle pane).

The bacterial abundance and correlation networks of the four communities changed significantly in NSCLC patients, especially core communities 1 and 2. All bacteria in community 1 showed decreased abundances in the NSCLC patients compared with the healthy subjects, e.g., *Sphingomonas* (*P* < 0.05) and *Bradyrhizobium* (*P* < 0.05) (Fig. 1B). Similarly, all bacteria in community 3 and most bacteria in community 4 (except genus *Prevotella 9*) also showed decreased abundances in the NSCLC patients compared with the healthy subjects, e.g., *Lactobacillus* (*P* < 0.01) and *Phascolarctobacterium* (*P* < 0.05) (Fig. 1D and E). Interestingly, most bacteria in community 2 (except genus *Haemophilus*) showed increased abundances in NSCLC patients compared with healthy subjects, e.g., *Neisseria* (*P* < 0.05), *Veillonella* (*P* < 0.01), *Prevotella 7* (*P* < 0.05), *Capnocytophaga* (*P* < 0.05), *Leptotrichia* (*P* < 0.01), *Porphyromonas* (*P* < 0.05), *Actinomyces* (*P* < 0.05), and *Granulicatella* (*P* < 0.01) (Fig. 1C). The correlation network of community 1 was less dense in NSCLC patients, whereas that of community 2 was denser compared with healthy subjects (Fig. 1A, S2). The decline of community 1 and dominance of community 2 in NSCLC patients could be further explained by the community degree counts. For example, the degree counts of community 1 decreased from 13.33 in healthy subjects to 11.67 in NSCLC patients (*P* = 0.2842), accompanied by an increase in degree counts of community 2 from 9.10 in healthy subjects to 12.59 in NSCLC patients (*P* = 0.0010) (Figure S3A, S3B). In addition, the degree counts of community 3 also decreased in NSCLC patients (from 3.50 to 3.00, *P* = 0.3521) (Figure S3C). These results suggest that different communities of lung mucosa-colonizing bacteria, especially core communities 1 and 2, play different roles in NSCLC carcinogenesis and progression.

**Fig. 2:**
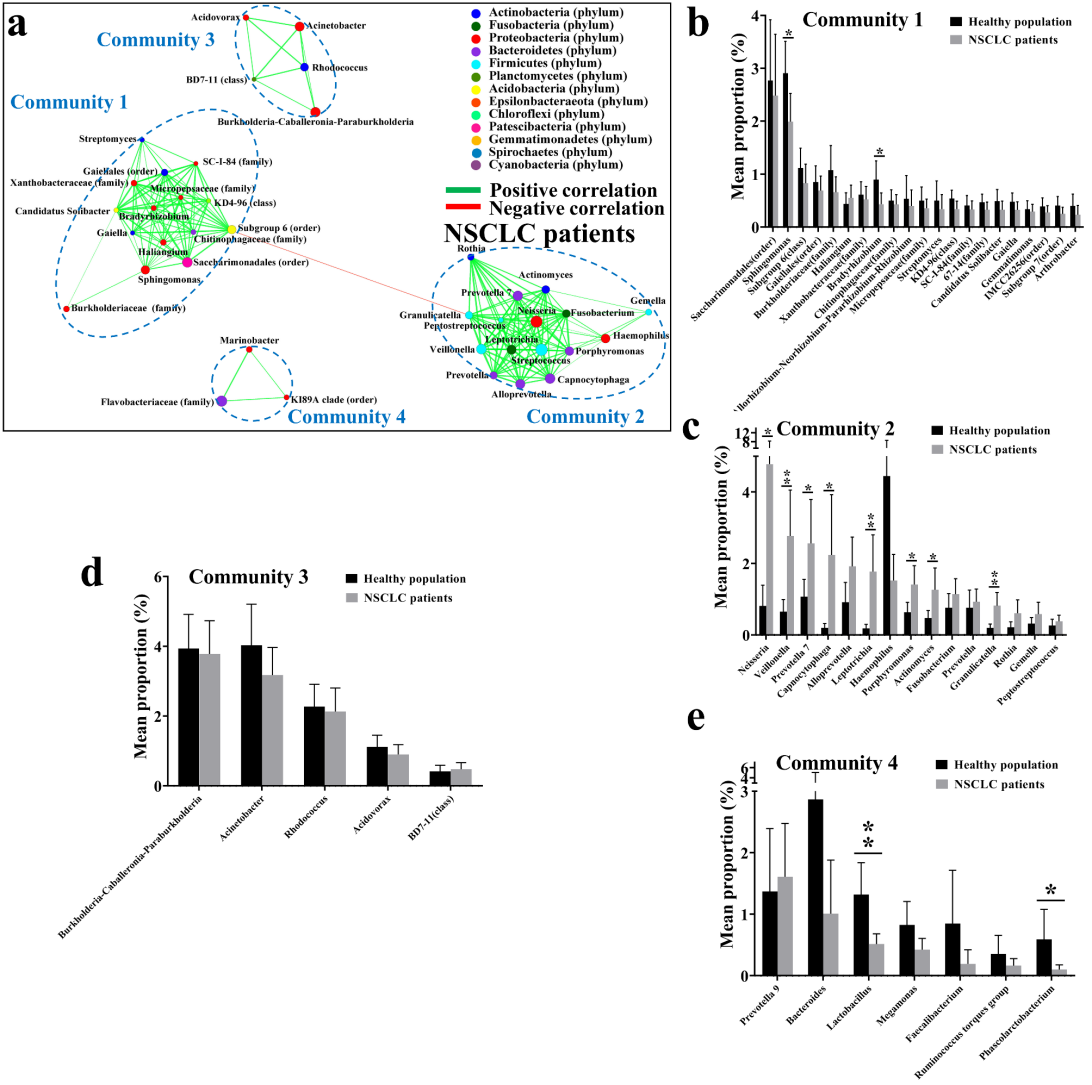
**Community structure of lung mucosa-colonizing bacteria and correlation between community members and immune cells in metastatic NSCLC patients.** (**A**) Correlation network of mucosa-colonizing bacteria in metastatic (n = 29) NSCLC patients. Correlation network was constructed based on Spearman rank correlation coefficients (∣Spearman Coef∣ ≥ 0.5, *P* < 0.05). Dotted lines encircle different bacterial communities containing relatively stable inner members that positively are correlated with each other. Node diameter is positively related to bacterial abundance. Node colors indicate different bacterial phyla. Lines connecting different nodes indicate positive (green) or negative (red) correlation between bacteria, and line diameter is positively related to correlation value. (**B**) Correlation heatmap between lung mucosa-colonizing bacteria and immune cells (as environmental factors) in peripheral venous blood of metastatic (n = 29) NSCLC patients. Correlations are positive (green) or negative (red) when R value is greater or less than 0, respectively. * 0.01 < *P* ≤ 0.05, ** 0.001 < *P* ≤ 0.01, *** *P* ≤ 0.001. Immune cells include neutrophils, monocytes, lymphocytes, eosinophils, and basophils. (**C**) Two-way correlation network between lung mucosa-colonizing bacteria and immune cells (as environmental factors) in peripheral venous blood of metastatic (n = 29) NSCLC patients. Correlation network was constructed based on Pearson rank correlation coefficients (∣Pearson Coef∣ ≥ 0.5, *P* < 0.05). Lines connecting different nodes indicate positive (green) or negative (red) correlation between bacteria and immune cells. Abundance comparison of community 1 **(D)** and 2 **(E)** members between metastatic (n = 29) and non-metastatic (n = 26) NSCLC patients. Y axis represents relative abundance percentage (mean proportion) of each bacterial genus in healthy populations and NSCLC patients. * 0.01 < *P* ≤ 0.05, confidence interval (CI) = 0.95. Correlation network and heatmap contain top 50 abundant bacteria. All bacteria were named to genus level unless noted otherwise in brackets

To investigate the characteristics of metastasis-associated community structure, we compared bacterial abundance, correlation network, and degree counts between the metastatic and non-metastatic NSCLC patients. Although both alpha and beta diversities of lung mucosa-colonizing bacteria did not significantly increase in the metastatic group (Figure S4), all bacteria in community 1 showed increased abundance in the metastatic group compared with the non-metastatic group (e.g., *Bradyrhizobium*, *P* < 0.05; *Streptomyces*, *P* < 0.05) (Fig. 2D). Similar abundance increases were observed in most community 4 bacteria (except *Lactobacillus*) (Figure S7B). Several members in community 2, e.g., genera *Prevotella 7*, *Alloprevotella*, *Porphyromonas*, *Prevotella*, *Granulicatella*, *Rothia*, *Gemella*, and *Peptostreptococcus*, also showed increased abundance in the metastatic NSCLC patients compared with non-metastatic group (Fig. 2E). Only bacteria in community 3 showed decreased abundances (Figure S7A). In addition, increased correlation network strength in community 1 was observed in the metastatic NSCLC patients (Fig. 2A) compared with the non-metastatic group (Figure S5). The degree counts in community 1 also showed a significant increase in the metastatic NSCLC patients (*P* = 0.0212) (Figure S8A). However, community 3 showed a decrease in degree counts in the metastatic group (*P* = 0.0182) (Figure S8C). As all community 2 bacteria showed increased abundance in NSCLC patients, and some were further increased in the metastatic NSCLC patients, we proposed that certain community 2 bacteria are positively and extensively correlated with NSCLC progression and metastasis incidence. In addition, as community 1 bacteria decreased in NSCLC overall, but increased in metastatic NSCLC patients, they may be considered as results, but not reasons of NSCLC progression and metastasis.

To further demonstrate the association between community structure and NSCLC metastasis incidence, we analyzed the abundances of community 1 and 2 members in metastatic SCC and AC, the two main subtypes of NSCLC. Consistent with the metastatic NSCLC patients, all community 1 bacteria showed increased abundances in the metastatic SCC patients compared with the non-metastatic group, especially genera *Bradyrhizobium*, *Streptomyces*, and *Gemmatimonas*, family *Burkholderiaceae*, and order *IMCC26256* (all *P* < 0.05) (Figure S10A). The degree count of community 1 also increased significantly in the metastatic SCC patients compared with the non-metastatic group (*P* < 0.0001) (Figure S11A). Some community 2 bacteria in metastatic SCC patients showed increased abundance, e.g., genera *Alloprevotella*, *Prevotella*, *Granulicatella*, *Gemella*, and *Peptostreptococcus* (Figure S10B). The degree count of community 2 also increased significantly (*P* < 0.0001) (Figure S11B). In AC patients, all community 1 bacteria showed increased abundances (Figure S13A). Furthermore, increased degree counts were observed of both community 1 (*P* < 0.0001) and community 2 (*P* < 0.0001) (Figure S14). Interestingly, community 2 bacteria with increased abundance changed to genera *Veillonella*, *Prevotella 7*, *Capnocytophaga*, *Actinomyces*, and *Rothia* in the metastatic AC patients, (Figure S13B), most of which showed decreased abundance in the metastatic SCC patients. This variation in community 2 bacteria supports the differences in host immune conditions found between SCC and AC patients in regard to tumor and cancer cell migration mechanisms.

We also investigated the community structure characteristics of NSCLC patients with different survival. Strikingly, NSCLC patients with poor survival (less than 3 months) exhibited low dense connections between different communities (Figure S16A), the connection densities increased when NSCLC patients with better survival (ranging from 3 to 8 months, Figure S17A), especially NSCLC patients with over 8 months survival (Figure S18A). It is worth mentioning that the inner connections within community 2 members gradually thinned when the NSCLC patients showed a longer survival. In addition, NSCLC patients with poor survival (less 3 months) exhibited significant and positive correlations between community 2 members and eosinophils, neutrophils, and monocytes (Figure S16B), when the other patients with longer survival did not exhibit such significant correlations anymore (Figure S17B, S18B). This correlation variation between different communities of NSCLC patients with poor or better survivals was similar to that of patients with or without metastasis.

### Association of Bacterial Community structure with Immune cells

We counted the numbers of five different leukocytes, including lymphocytes, neutrophils, eosinophils, monocytes, and basophils, in the peripheral blood of NSCLC patients and compared them between the non-metastatic and metastatic NSCLC patients (Table 2). Except for basophils, the lymphocytes, neutrophils, eosinophils, and monocytes all showed an increase in cell number in the metastatic NSCLC patients compared with the non-metastatic group. These core immune cells showed an increased concentrations in the metastatic group compared with the non-metastatic group, such as lymphocytes (*P* = 0.0374) and neutrophils (*P* = 0.0027).

**Table 2:** Immune factor concentrations and immune cell densities in peripheral blood of NSCLC (including SCC and AC) patients with or without cancer cell metastasis

Index	NSCLC		SCC		AC		
	No met	Met	No met	Met	No met		Met
	(n = 26)	(n = 29)	(n = 20)	(n = 11)	(n = 6)		(n = 18)
*Lymphocyte*							
Average	1.30	1.61	1.32	1.92	1.27		1.42
SD	0.52	0.55	0.57	0.31	0.32		0.59
Lower 95% CI	1.09	1.40	1.05	1.71	0.89		1.12
Upper 95% CI	1.51	1.82	1.58	2.12	1.56		1.71
*P* value	0.0374		0.0033		0.4650		
*Monocyte*							
Average	0.56	0.60	0.51	0.59	0.70		0.66
SD	0.23	0.25	0.22	0.28	0.20		0.21
Lower 95% CI	0.47	0.50	0.41	0.30	0.48		0.56
Upper 95% CI	0.65	0.69	0.62	0.67	0.91		0.77
*P* value	0.5470		0.7695		0.7230		
*Neutrophils*							
Average	5.51	7.52	4.90	5.21	5.41		6.60
SD	2.23	2.48	1.80	1.98	2.45		1.34
Lower 95% CI	4.61	6.57	4.06	3.89	2.85		5.94
Upper 95% CI	6.41	8.46	5.74	6.54	7.98		7.27
*P* value	0.0027		0.6588		0.1421		
*Eosinophils*							
Average	0.14	0.23	0.14	0.24	0.17		0.23
SD	0.12	0.26	0.12	0.10	0.14		0.32
Lower 95% CI	0.09	0.13	0.08	0.18	0.02		0.07
Upper 95% CI	0.19	0.33	0.19	0.31	0.32		0.39
*P* value	0.1113		0.0163		0.6682		
*Basophils*							
Average	0.04	0.02	0.04	0.01	0.03	0.03	
SD	0.08	0.02	0.09	0.01	0.01	0.03	
Lower 95% CI	0.01	0.01	0.01	0.01	0.01	0.02	
Upper 95% CI	0.07	0.03	0.09	0.02	0.04	0.04	
*P* value	0.2893		0.2772		0.7798		

Note: Data are in 10^9^ cells/L peripheral venous blood. NSCLC, non-small cell lung adenocarcinoma; SCC, lung squamous cell carcinoma; AC, lung adenocarcinoma; No met, no metastasis

Leukocytes, such as lymphocytes and neutrophils, circulating in peripheral blood can infiltrate tumor tissues and regulate NSCLC progression and metastasis [29, 30]. However, abnormal bacterial colonization in lower airways can induce extensive accumulation of these leukocytes in host bronchoalveolar lavage fluid [31]. Therefore, we performed Pearson correlation analysis on peripheral blood leukocytes and lung mucosa-colonizing bacteria in the metastatic and non-metastatic NSCLC patients. Analysis revealed that three types of leukocytes, i.e., eosinophils, neutrophils, and monocytes, were significantly and positively correlated with eight genera (i.e., *Haemophilus*, *Granulicatella*, *Gemella*, *Streptococcus*, *Porphyromonas*, *Alloprevotella*, *Prevotella*, and *Peptostreptococcus*) (Fig. 2B C). Interestingly, all eight genera belonged to community 2 and showed increased abundances in metastatic NSCLC patients (Fig. 2E). Of note, no significant and negative correlations were found between leukocytes and any bacterium in the metastatic NSCLC patients (Fig. 2B). In the non-metastatic NSCLC patients, however, eosinophils, neutrophils, and monocytes were not significantly and positively correlated with community 2 members. In detail, the relationship between leukocytes, including eosinophils and monocytes, and community 2 bacteria shifted to a negative (albeit non-significant) correlation. Eosinophils also showed a significant and positive correlation with community 3 (genera *Acidovorax* and *Cutibacterium*) and community 4 members (genus *Marinobacter*, order *KI89A clade*, and family *Flavobacteriaceae*). Neutrophils also showed a significant, but negative correlation with community 3 members (genera *Acidovorax, Cutibacterium*, *Acinetobacter* and *Burkholderia-Caballeronia-Paraburkholderia* (*Burkholderia*)). Monocytes showed a significant and negative correlation with community 4 members (genus *Bacteroides*), although the significant and positive correlation of monocytes with community 2 bacteria (genus *Granulicatella*) remained (Figure S6). In addition, basophils significantly and positively correlated with community 3 members (order *Chloroplast* and genus *Corynebacterium 1*) of metastatic group (Fig. 2B C), but with community 2 members (genera *Actinomyces*, *Leptotrichia*, *Campylobacter*, *Capnocytophaga*, *Streptococcus*, *Veillonella*, *Porphyromonas* and family *Actinomycetaceae*) of the non-metastatic group (Figure S6). Unexpectedly, lymphocytes, which have received considerable attention in tumor progression and metastasis [29], showed the least correlations with lung mucosa-colonizing bacteria. Lymphocytes were only significantly positively correlated with genus *Prevotella 9* (community 4 member) in the metastatic group and significantly negatively correlated *Sphingomonas* (community 1 member) in the non-metastatic group (Fig. 2B, S6). These results indicate that lymphocytes may not be the core responders to the colonization of lung mucosal-bacterial communities, at least at the level of total lymphocytes.

To further confirm the significant and positive correlations between the three types of leukocytes (eosinophils, neutrophils, and monocytes) and lung mucosa-colonizing community 2 members in metastatic NSCLC patients, we performed correlation analysis between bacterial abundance and leukocytes in the metastatic SCC and AC groups. Similarly, eosinophils, neutrophils, and monocytes showed significant and positive correlations with community 2 members (genera *Gemella*, *Prevotella 6*, *Prevotella*, *Actinomyces*, *Alloprevotella*, *Haemophilus*, *Peptostreptococcus*, *Granulicatella*, *Prevotella 2*, *Streptococcus*, and *Porphyromonas*) in metastatic SCC patients (Figure S9A, S9C), all of which showed increased abundances compared with the non-metastatic group (Figure S10). No significant negative correlations existed between leukocytes and lung mucosa-colonizing bacteria in the metastatic SCC patients. The non-metastatic SCC patients also showed significant positive correlations between eosinophils and community 1 members, significant negative correlations between neutrophils and community 3 members, and significant positive correlations between basophils and community 2 members (Figure S9B, S9D). In AC patients, although the correlations between leukocytes in peripheral blood and lung mucosa-colonizing bacteria were largely reduced (Figure S12), our results still showed that the strong positive correlation between eosinophils, neutrophils, and monocytes and community 2 members is a basic characteristic of the correlation network in the metastatic group (Fig. 4C right pane), and this principle is applicable for all metastatic NSCLC patients.

### Association of Bacterial Community structure with Cancer markers

**Fig. 3:**
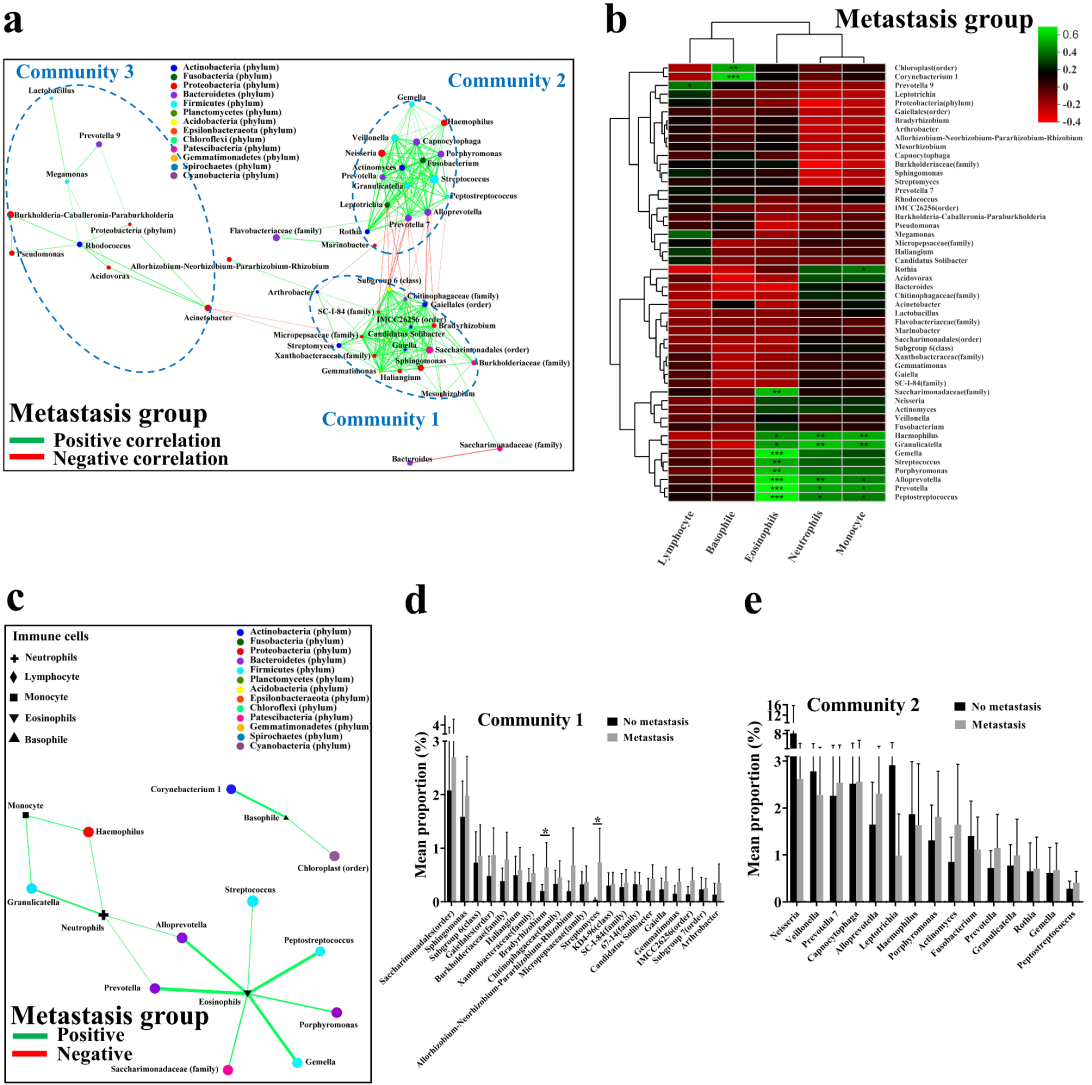
**Correlation between community members of lung mucosa-colonizing bacteria and cancer markers in metastatic and non-metastatic NSCLC patients.** Correlation heatmap between lung mucosa-colonizing bacteria and cancer markers (as environmental factors) in peripheral venous blood of metastatic (**A)** and non-metastatic (**B)** NSCLC patients. Correlations are positive (green) or negative (red) when R value is greater or less than 0, respectively. * 0.01 < *P* ≤ 0.05, ** 0.001 < *P* ≤ 0.01, *** *P* ≤ 0.001. Cancer markers include carcinoembryonic antigen (CEA), neuron-specific enolase (NSE), cytokeratin-19-fragment (CYFRA21-1), carbohydrate antigen 125 (CA125), and squamous cell carcinoma antigen (SCCA). Two-way correlation network between lung mucosa-colonizing bacteria and cancer markers (as environmental factors) in peripheral venous blood of metastatic (**C)** and non-metastatic (**D)** NSCLC patients. Correlation network was constructed based on Pearson rank correlation coefficients (∣Pearson Coef∣ ≥ 0.5, *P* < 0.05). Lines connecting different nodes indicate positive (green) or negative (red) correlation between bacteria and immune cells. For metastatic and non-metastatic NSCLC patients n = 31 and 22, respectively. Correlation network and heatmap contain top 50 abundant bacteria. All bacteria were named to genus level unless noted otherwise in brackets

Several markers in peripheral blood are routinely applied for NSCLC diagnosis, including squamous cell carcinoma antigen (SCCA), neuron-specific enolase (NSE), carcinoembryonic antigen (CEA), cytokeratin 19 fragment (CYFRA21-1), and carbohydrate antigen 125 (CA125) [32, 33]. Several of these markers can also predict metastasis incidence [34, 35]. Thus, the correlations between cancer markers and lung mucosa-colonizing bacteria could provide an applicable and reliable strategy for clinical diagnosis of early metastasis in NSCLC patients. We tested the concentrations of five different cancer markers above, in the peripheral blood of NSCLC patients and compared them between the non-metastatic and metastatic NSCLC patients (Table 3). Here, we observed significant and positive correlations between NSE and community 3 members (genera *Bacteroides*, *Acidovorax*, *Burkholderia*, *Rhodococcus*, and *Acinetobacter*) and SCCA and community 1 members (genera *Gemmatimonas*, *Haliangium*, *Candidatus Solibacter*, *Gaiella*, and family *Micropepsaceae*) in metastatic NSCLC patients. Other markers, CYFRA21-1, CEA, and CA125, showed significant and positive correlations with genus *Rothia* (community 2) and family *Chitinophagaceae* (community 1) (Fig. 3A, C). In the non-metastatic NSCLC patients, however, these correlations all disappeared. Instead, CYFRA21-1 showed a significant and positive correlation with community 2 (genera *Alloprevotella*, *Prevotella 7*, and *Prevotella 6*) and community 4 members (order *KI89A clade*, order *Flavobacteriaceae*, and family *Marinobacter*). CEA and CA125 showed significant and positive correlations with community 3 members (genera *Bacteroides*, *Burkholderia*, *Cloacibacterium*, *Acinetobacter*, order *Subgroup 2*, and genus *Rhodococcus*, order *Chloroplast*) (Fig. 3B, D). Interestingly, no significant negative correlations existed between cancer markers and lung mucosa-colonizing bacteria. In addition, the correlations between lung mucosa-colonizing bacteria and cancer markers were not tight or logical compared with the correlations between lung mucosa-colonizing bacteria and immune cells in peripheral blood.

**Table 3:** Typical cancer marker concentrations in peripheral blood of NSCLC (including SCC and AC) patients with or without cancer cell metastasis

Index	NSCLC		SCC		AC	
	No met	Met	No met	Met	No met	Met
	(n = 22)	(n = 31)	(n = 18)	(n = 13)	(n = 4)	(n = 18)
*CEA*						
Average	3.95	353.20	4.20	49.81	2.80	572.40
SD	3.92	914.80	4.30	82.16	0.47	1 162.00
Lower 95% CI	2.21	17.68	2.06	0.16	2.05	-5.62
Upper 95% CI	5.68	688.80	6.34	99.46	3.55	1 150.00
Significance	No		*		No	
*NSE*						
Average	23.10	30.51	24.51	21.43	16.76	37.06
SD	22.12	32.56	24.35	8.86	1.30	41.31
Lower 95% CI	13.29	18.56	12.40	16.07	14.69	16.52
Upper 95% CI	32.91	42.45	36.62	26.79	18.82	57.61
Significance	No		No		No	
*CYFRA21-1*						
Average	10.15	19.85	11.26	12.61	5.17	25.07
SD	10.31	27.13	11.12	6.53	1.78	34.65
Lower 95% CI	5.58	9.89	5.73	8.66	2.33	7.84
Upper 95% CI	14.72	29.80	16.78	16.55	8.00	8.17
Significance	No		No		No	
*CA125*						
Average	57.62	733.40	65.87	63.40	20.50	1 217.00
SD	74.03	2209.0	79.82	66.04	4.04	2 831.00
Lower 95% CI	24.80	-76.85	26.18	23.49	14.07	-190.70
Upper 95% CI	90.45	1 544.0	105.60	103.30	26.93	2625
Significance	No		No		No	
*SCCA*						
Average	1.87	1.48	2.04	2.16	1.10	0.99
SD	1.68	1.47	1.82	1.76	0.12	1.02
Lower 95% CI	1.13	0.94	1.14	1.10	0.92	0.48
Upper 95% CI	2.61	2.02	2.95	3.23	1.28	1.50
Significance	No		No		No	

Note: Data are in ng/mL peripheral venous blood, except for CA125 in units/mL. AFP, alpha fetoprotein; CEA, carcinoembryonic antigen; NSE, neuron-specific enolase; CYFRA21-1, cytokeratin-19-fragment; CA125, carbohydrate antigen 125; SCCA, squamous cell carcinoma antigen

### Effects of lung Bacteria on NSCLC Cell Migration

**Fig. 4:**
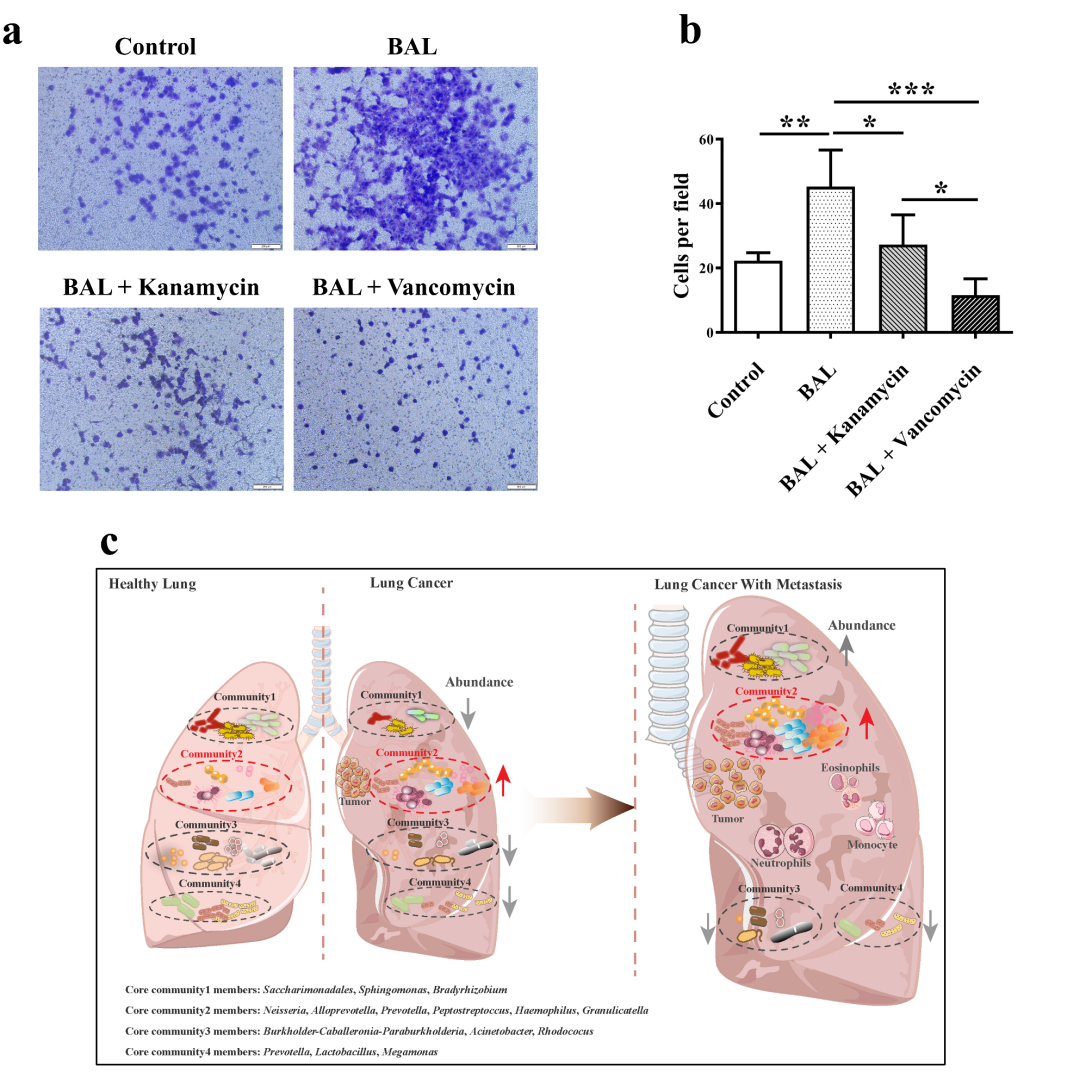
**Effects of bronchoalveolar lavage fluid from AC patients on NSCLC cell migration.** H1299 human NSCLC cell line with high metastasis potential was seeded into a 24-well transwell plate. At 24 h after seeding, bronchoalveolar lavage (BAL) fluid (50 µL) from AC patient 1# was added to the cell culture well. Vancomycin (2.5 µg/L) and kanamycin (5 µg/L) were also added for BAL + vancomycin group and BAL + kanamycin group, respectively. At 48 h after BAL and antibiotic management, migrated cells were stained (**A**) and counted (**B**). (**C**) Schematic representation of abundance variation of lung mucosa-colonizing bacteria communities and surrounding immune cells in different NSCLC stage. Left pane: lung mucosa-colonizing bacteria of healthy population were basically divided into four communities (Community 1, 2, 3, 4); Middle pane: member abundances of most communities, except community 2, increase in NSCLC patients; Right pane: most members of community 2 further increase their abundances in NSCLC patients with metastasis, and they positively correlate with immune cells, such as eosinophils, neutrophils and monocytes

To further confirm the association between NSCLC metastasis and lung mucosa-colonizing bacteria, we performed cell migration assays using the human NSCLC cell line H1299 and AC bronchoalveolar lavage fluid containing lung mucosa-colonizing bacteria. Results showed that AC bronchoalveolar lavage fluid significantly increased cell migration number (22.25 to 45.25, *P* < 0.01). Furthermore, we applied two antibiotics, vancomycin and kanamycin, which are frequently used in clinical practice for the management of bacterial lung infection [36, 37], to treat the H1299 cells. Surprisingly, NSCLC cell migration was significantly reduced by both vancomycin (45.25 to 11.50, *P <* 0.001) and kanamycin (45.25 to 27.25, *P* < 0.05) (Fig. 4). This inhibition of NSCLC cell migration by antibiotics was reproduced by the AC bronchoalveolar lavage fluid from different AC patients (Figure S15). Although this migration assay did not consider peripheral blood immune cells in NSCLC cell metastasis incidence, antibiotics targeting specific lung mucosa-colonizing bacteria that are positively related to NSCLC cell migration could be of interest for the management of NSCLC patients with metastasis.

## Discussion

Greathouse et al. show an increased alpha diversity (both Chao and Simpson indexes) in neoplastic lung tissue compared with normal lung [38]. However, our study showed a decreased diversity (both Chao and Shannon indexes) in lung mucosa of NSCLCL patients compared with which of healthy population, that seeming contrary with Greathouse’s study. Actually, that is reasonable because we collected lung mucosa samples of healthy population and NSCLC patients, Greathouse et al. used neoplastic lung tissue for 16 S rRNA sequencing. Bacterial colonization condition is completely different from lung tissue inner to lung mucosa. Furthermore, healthy lungs allow the colonization of non-pathogenic bacteria, that forming a stable colonizing homeostasis on lung mucosa. Neoplastic lung tissue, however, suppresses local immune activity and leaves chances for (pathogenic) bacteria entrance, that inducing mucosa-colonizing bacteria invasion into lung tissue inner and consequent increased bacterial burden. Therefore, the mounts of lung mucosa-colonizing bacteria relatively decrease, because many of them invade into lung tissue and tumor nidus where host immune is greatly suppressed and nutrition supply is rich.

The lung mucosa-colonizing bacteria of healthy subjects and NSCLC patients formed a stable bacterial ecosystem, which could be divided into four communities. Core communities 1 and 2 had different performances on bacterial abundance, correlation network and correlation model with immune cells and cancer markers in peripheral blood in metastatic NSCLC patients. Specifically, community 2 members were prominently involved in NSCLC progression, including metastasis. This distinct and prominent performance of community 2 members on correlating with clinical features could provide a novel strategy for predicting early metastasis incidence, which is difficult to detect under normal clinical methods.

Certain bacteria inside lung tumors promote disease progression [39] and these bacteria feature lung tumor-specific community structures [40]. While such research suggests the involvement of lung tumor-colonizing bacteria and related bacterial communities in lung cancer development, the correlations between community structure of lung mucosa-colonizing bacteria and cancer cell metastasis remain unclear.

In this study, almost all community 2 bacteria showed increased abundances in the NSCLC patients compared with the healthy subjects. In addition, most of these bacteria also showed increased abundance in the metastatic NSCLC patients compared with the non-metastatic patients, e.g., genera *Alloprevotella*, *Porphyromonas*, *Actinomyces*, *Prevotella*, *Granulicatella*, *Rothia*, *Gemella*, and *Peptostreptococcus*. Although these bacteria are all reportedly associated with lung cancer [41], no study has previously revealed the correlation between these bacteria and immune cells in peripheral blood in a community-dependent manner. Here, these bacteria were significantly correlated with leukocytes (eosinophils, neutrophils, and monocytes) in the metastatic NSCLC patients. The increased abundances of these bacteria and significant correlations with the three types of leukocytes were also observed in the metastatic SCC and AC patients.

Besides, we also noticed the metastatic group did not show any significant negative correlation, while the non-metastatic group exhibited the significant negative correlation between neutrophils and community 3 members. Although community 1 bacteria showed increased abundances in the metastatic group, they showed no significant correlation with leukocytes, and thus appear to be exhibitors not drivers of NSCLC metastasis. Due to the significant correlation between specific leukocytes and community 2 bacteria, our research suggests that specifically targeting community 2 members instead of broad-spectrum antibiotics could hold considerable potential in the prevention of metastasis after NSCLC resection.

Examination of peripheral blood-containing cancer markers is a common strategy for NSCLC diagnosis in clinical practice [32]. Correlation analysis of these markers with lung mucosa-colonizing bacterial communities should improve NSCLC diagnosis accuracy, especially metastasis, as cancer cell death and marker protein infiltration in peripheral blood are tightly associated with immune cells around tumors [42] and tumor-infiltrating immune cell profiles are tightly assocaited with local bacteria[5, 6]. Our study indicated that cancer markers, e.g., NSE and SCCA, were significantly and positively correlated with community 1 and 3 members, but not community 2 members in the metastatic NSCLC patients. Notably, no significant negative correlations between bacteria and cancer markers were observed in either the metastatic or non-metastatic groups, different to the correlation found between bacteria and immune cells. This phenotype could be explained by the increasing concentration of cancer markers as NSCLC deteriorates.

We applied a cell migration assay to investigate the effects of lung mucosa-colonizing bacteria on the incidence of NSCLC metastasis and revealed that alveolar lavage fluid containing lung mucosa-colonizing bacteria directly promoted NSCLC cell migration. This assay does needs improvement regarding the leukocyte presence and oxygen concentration in the cell incubator, i.e., the oxygen balance in the cell incubator differs from that found in the lower airways of humans and most anaerobic bacteria colonizing lung mucosa die. Besides, the data from this assay were not potent enough in exhibiting the correlation of metastasis incidence with immune cells, because the microenvironment of blood is largely different from alveolar lavage fluid, that containing very few immune cells. However, our results also emphasize that aerobic bacteria or facultative anaerobes may play a critical and independent role in the incidence of NSCLC metastasis.

In addition, several questions remain to be addressed. During NSCLC progression, especially during metastasis, both leukocyte profiles and bacterial community structures continue to change [29, 43]. Therefore, their correlation at different NSCLC stages also needs to be explored. The leukocytes used in our study were obtained from peripheral blood; however, leukocytes inside tumor tissues may be a better option to analyze correlations with lung mucosa-colonizing bacteria. Recent studies have summarized the great advantages of single-cell spatial omics in identifying novel predictive biomarkers and precision-medicine targets [44]. Therefore, we suggest that combining spatial omics and bacterial community structure may be a potential strategy for early metastasis prediction.

## Conclusions

Our study revealed that lung mucosa-colonizing bacteria were comprised of four communities, each with their own members. The bacterial community structure was found in both healthy and diseased participants. Several community 2 bacteria (genera *Alloprevotella*, *Porphyromonas*, *Actinomyces*, *Prevotella*, *Granulicatella*, *Rothia*, *Gemella*, and *Peptostreptococcus*) showed increased abundances not only in NSCLC patients, but also in the metastatic group (Fig. 4C). Furthermore, these bacteria showed significant positive correlations with eosinophils, neutrophils, and monocytes in the metastatic group, unlike that found in the non-metastatic group. The correlation between lung mucosa-colonizing bacteria and cancer markers in peripheral blood also changed from the metastatic to non-metastatic NSCLC patients. These differences in the correlation between lung mucosa-colonizing bacteria and immune cells and cancer markers in peripheral blood hold great promise in the diagnosis of NSCLC and related metastasis. We also found that lung mucosa-colonizing bacteria directly promoted NSCLC cell metastasis. Therefore, we suggest alteration of the micro-environment has a great value of inhibiting NSCLC metastasis incidence.

## Electronic supplementary material

Below is the link to the electronic supplementary material.

Supplementary Material 1

## Data Availability

The sequencing data generated in this study were submitted to SRA under BioProject accession number: PRJNA667552 (https://www.ncbi.nlm.nih.gov/bioproject/ PRJNA667552). The sequencing data were analyzed on the online platform of Majorbio Cloud Platform (www.majorbio.com) [23].

## Abbreviations

NSCLC: non-small cell lung cancer
AC: adenocarcinoma
SCC: squamous cell carcinoma
CEA: carcinoembryonic antigen
CT: computed tomography
TTF-1: thyroid transcription factor-1
NMRI: nuclear magnetic resonance imaging
BAL: bronchoalveolar lavage
SCCA: squamous cell carcinoma antigen
NSE: neuron-specific enolase
CEA: carcinoembryonic antigen
CYFRA21-1: cytokeratin 19 fragment
CA125: carbohydrate antigen 125
