# Supplementary material for: Correlation analysis of lung mucosa-colonizing bacteria with clinical features reveals metastasis-associated bacterial community structure in non-small cell lung cancer patients
Source: Respir Res. 2023 May 11;24:129. doi: 10.1186/s12931-023-02420-7 (PMC10176848; doi:10.1186/s12931-023-02420-7)
Supplement: Supplementary file 1 — Supplementary Material 1 [file 12931_2023_2420_MOESM1_ESM.docx]

**Supplementary Figures**


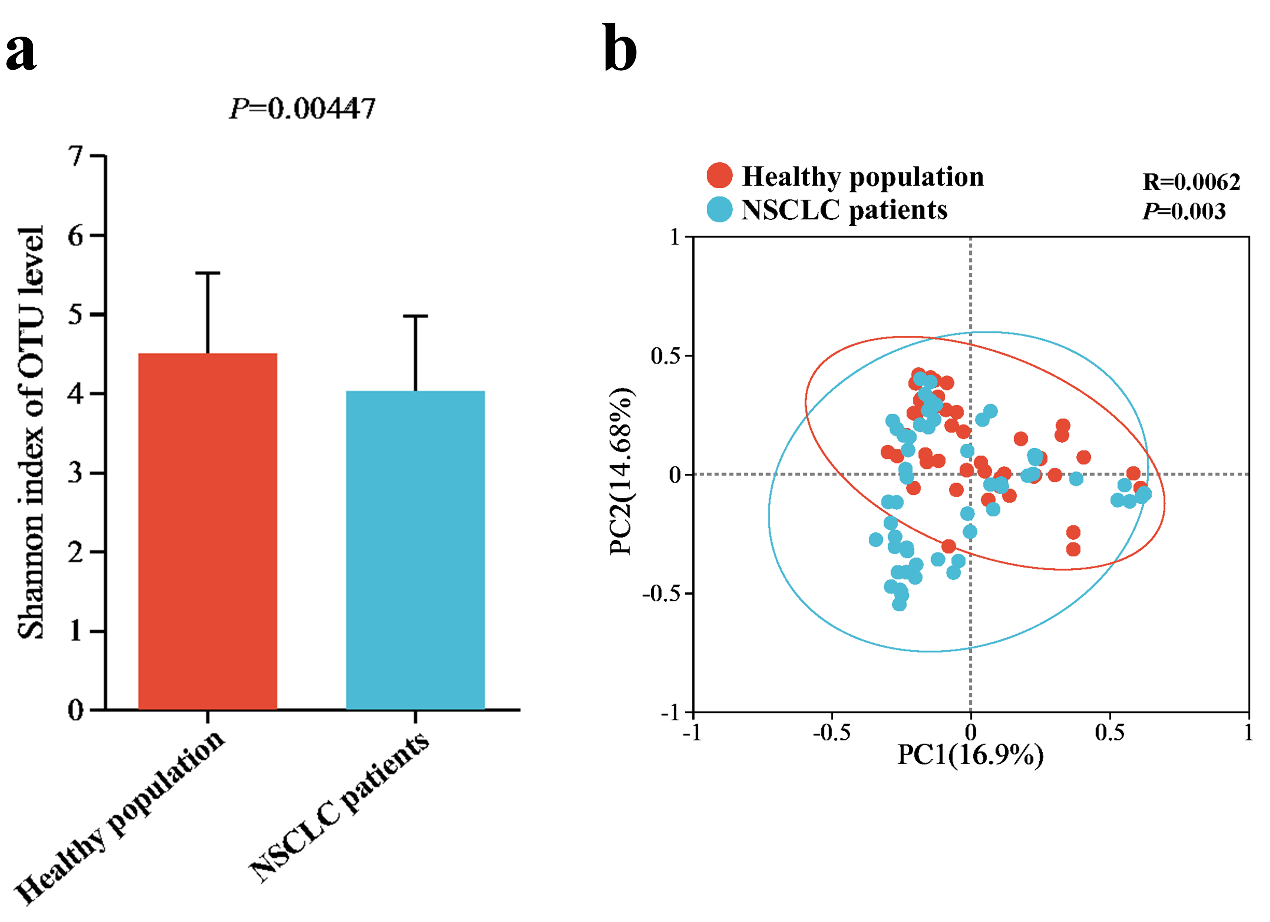


**Figure S1. Alpha and beta diversity of lung mucosa-colonizing bacterial in healthy population (n = 50) and NSCLC patients (n = 57)**. Decreased alpha diversities of NSCLC patient’s lung mucosa-colonizing bacteria were shown by Shannon index (**A**), compared with healthy population. The composition variation of lung mucosa-colonizing bacteria between healthy population and NSCLC patients was shown by principal co-ordinates analysis (PCoA) (**B**). PC1 and PC2 = principal components 1 and 2.


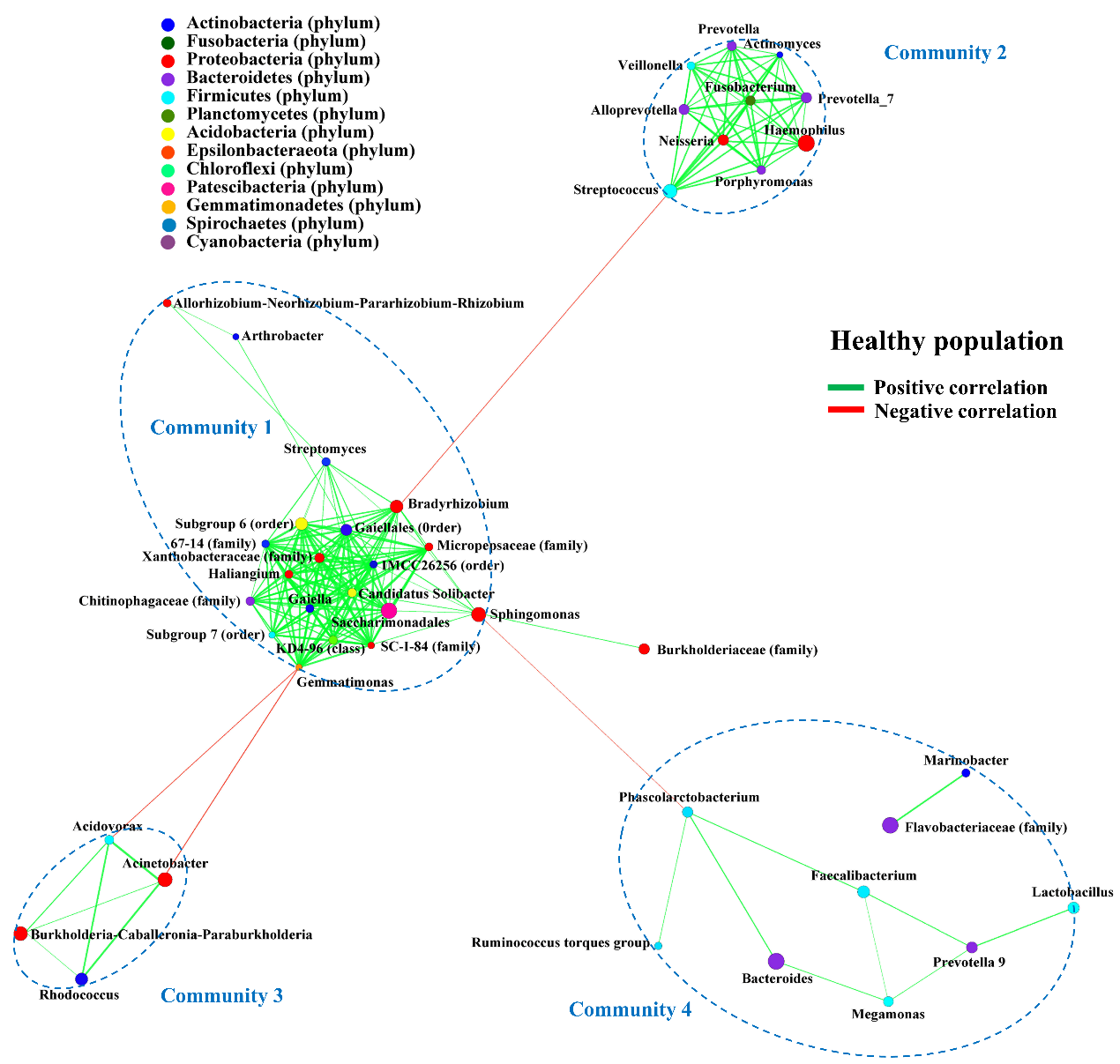


**Figure S2. Community structure of lung mucosa-colonizing bacteria in healthy population (n = 50)**. Correlation network was constructed based on Spearman rank correlation coefficients (∣Spearman Coef∣ ≥ 0.5, *P* < 0.05). Dotted lines encircle different bacterial communities containing relatively stable inner members that are positively correlated with each other. Node diameter is positively related to bacterial abundance. Node colors indicate different bacterial phyla. Lines connecting different nodes indicate positive (green) or negative (red) correlation between bacteria, and line diameter is positively related to correlation value. Correlation network and heatmap contain top 50 abundant bacteria. All bacteria were named to genus level unless noted otherwise in brackets.


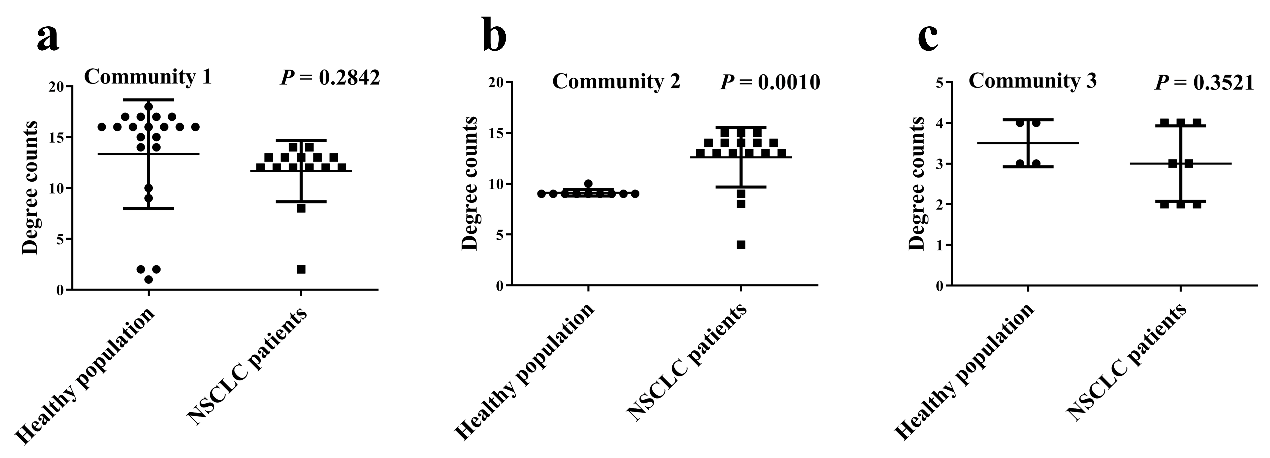


**Figure S3. Degree of lung mucosa-colonizing bacteria in healthy populations and NSCLC patients.** Comparison of degrees in community 1 **(A)** and 2 **(B)** of healthy populations (n = 50) and NSCLC patients (n = 57). Confidence interval (CI) = 0.95.


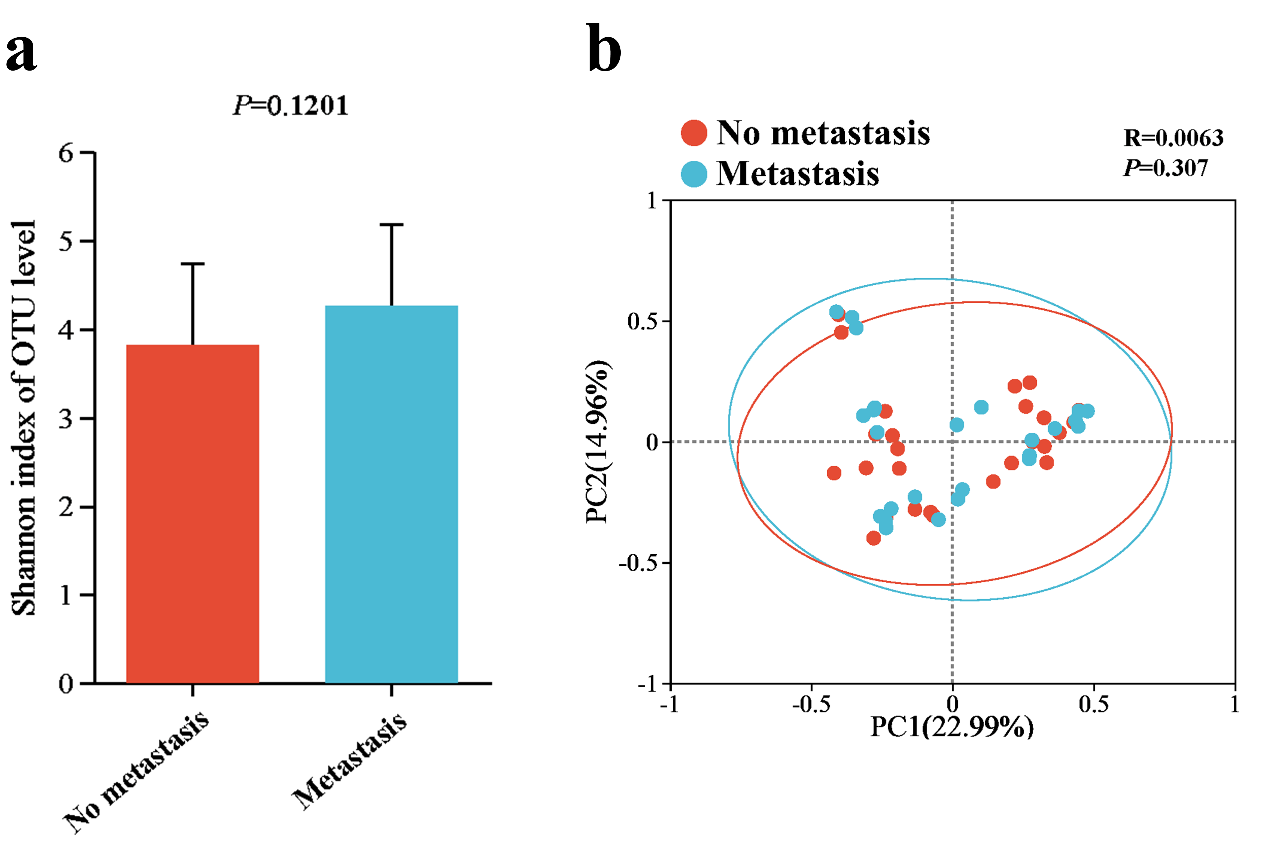


**Figure S4. Alpha and beta diversity of lung mucosa-colonizing bacteria in both no metastasis group (n = 26) and metastasis group (n = 29) of NSCLC patients**. Increased alpha diversities of lung mucosa-colonizing bacteria in NSCLC patient with metastasis were shown by Shannon index (**A**), compared with NSCLC patient without metastasis. The community composition variation of lung mucosa-colonizing bacteria within NSCLC patients with or without metastasis was shown by Principal co-ordinates analysis (PCoA) (**B**). PC1 and PC2 = principal components 1 and 2.


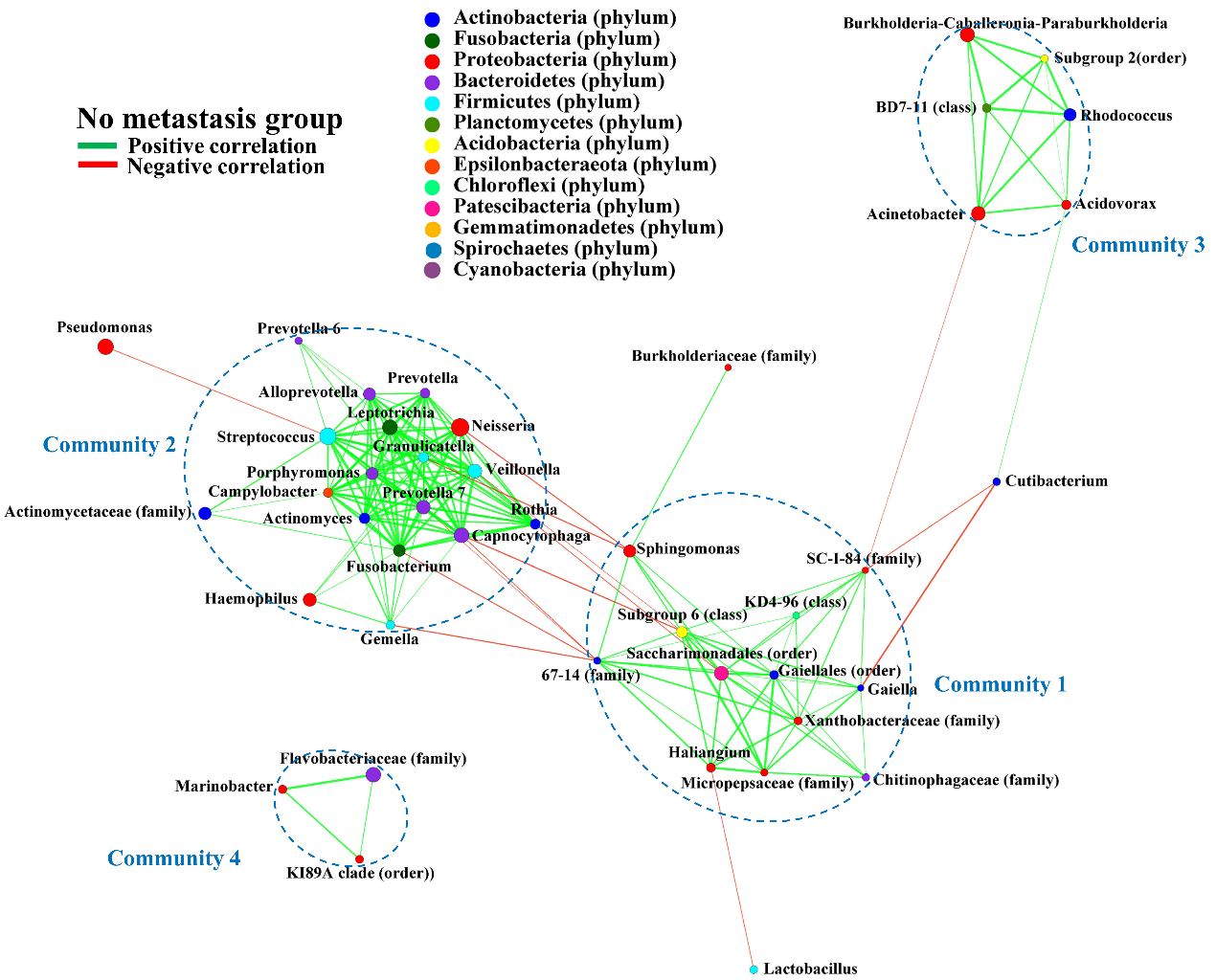


**Figure S5. Community structure of lung mucosa-colonizing bacteria in non-metastatic NSCLC patients**. Correlation network was constructed based on Spearman rank correlation coefficients (∣Spearman Coef∣ ≥ 0.5, *P* < 0.05). Dotted lines encircle different bacterial communities containing relatively stable inner members that are positively correlated with each other. Node diameter is positively related to bacterial abundance. Node colors indicate different bacterial phyla. Lines connecting different nodes indicate positive (green) or negative (red) correlation between bacteria, and line diameter is positively related to correlation value. For non-metastatic NSCLC patients n = 33. Correlation network and heatmap contain top 50 abundant bacteria. All bacteria were named to genus level unless noted otherwise in brackets.


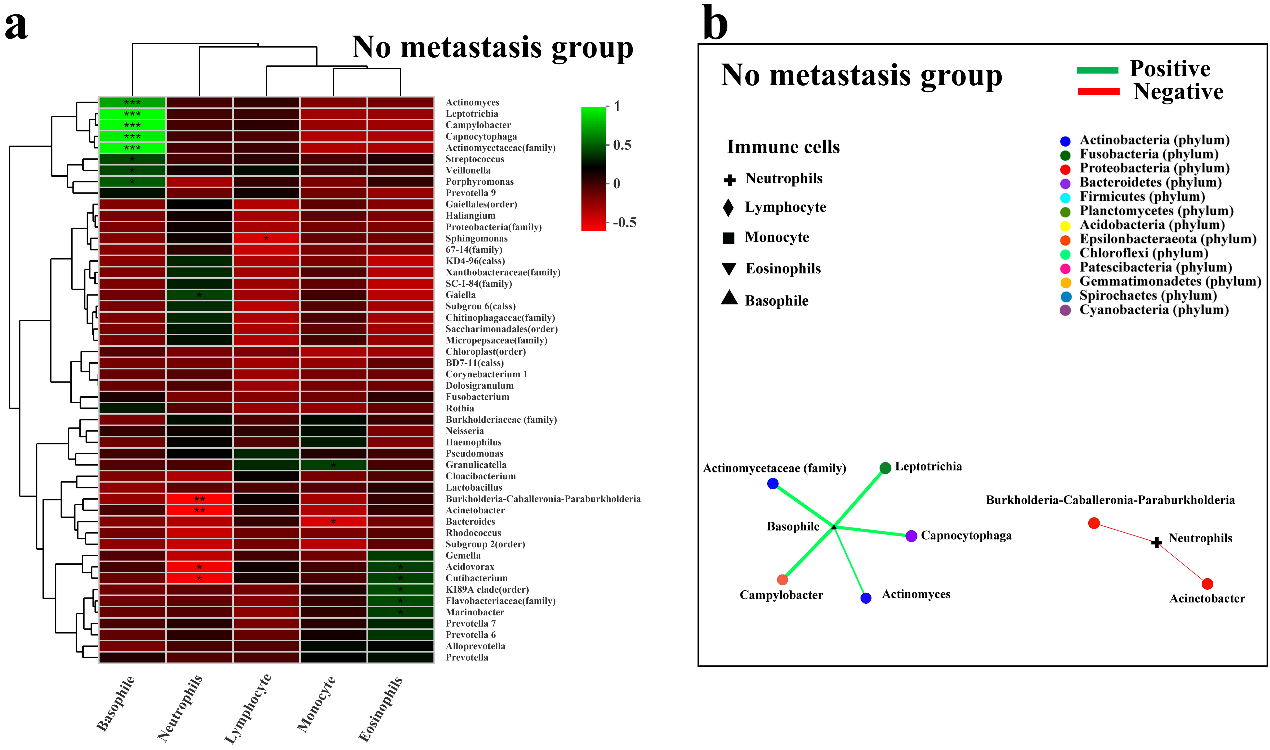


**Figure S6. Correlation between community members of lung mucosa-colonizing bacteria and immune cells in non-metastatic NSCLC patients**. (**A)** Correlation heatmap between lung mucosa-colonizing bacteria and immune cells (as environmental factors) in peripheral venous blood of metastatic (n = 29) NSCLC patients. Correlations are positive (green) or negative (red) when R value is greater or less than 0, respectively. * 0.01 < *P* ≤ 0.05, ** 0.001 < *P* ≤ 0.01, *** *P* ≤ 0.001. Immune cells include neutrophils, monocytes, lymphocytes, eosinophils, and basophils. **(B)** Two-way correlation network between lung mucosa-colonizing bacteria and immune cells (as environmental factors) in peripheral venous blood of metastatic (n = 26) NSCLC patients. Correlation network was constructed based on Pearson rank correlation coefficients (∣Pearson Coef∣ ≥ 0.5, *P* < 0.05). Lines connecting different nodes indicate positive (green) or negative (red) correlation between bacteria and immune cells. Correlation network and heatmap contain top 50 abundant bacteria. All bacteria were named to genus level unless noted otherwise in brackets.


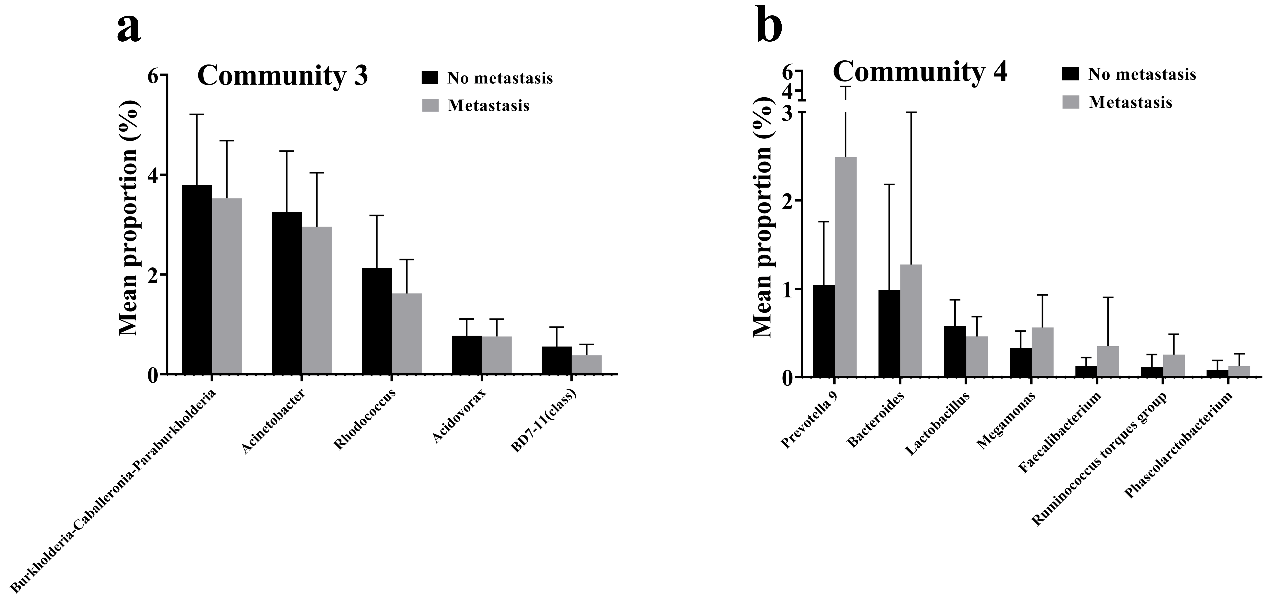


**Figure S7. Abundance comparison of community 3 (A) and 4 (B) bacteria between metastatic and non-metastatic NSCLC patients.** Y axis represents relative abundance percentage (mean proportion) of each bacterial genus in metastatic (n = 29) and non-metastatic (n = 26) NSCLC patients. * 0.01 < *P* ≤ 0.05, ** 0.001 < *P* ≤ 0.01, confidence interval (CI) = 0.95. Comparison of bacterial abundance of top 50 abundant bacteria. All bacteria were named to genus level unless noted otherwise in brackets.


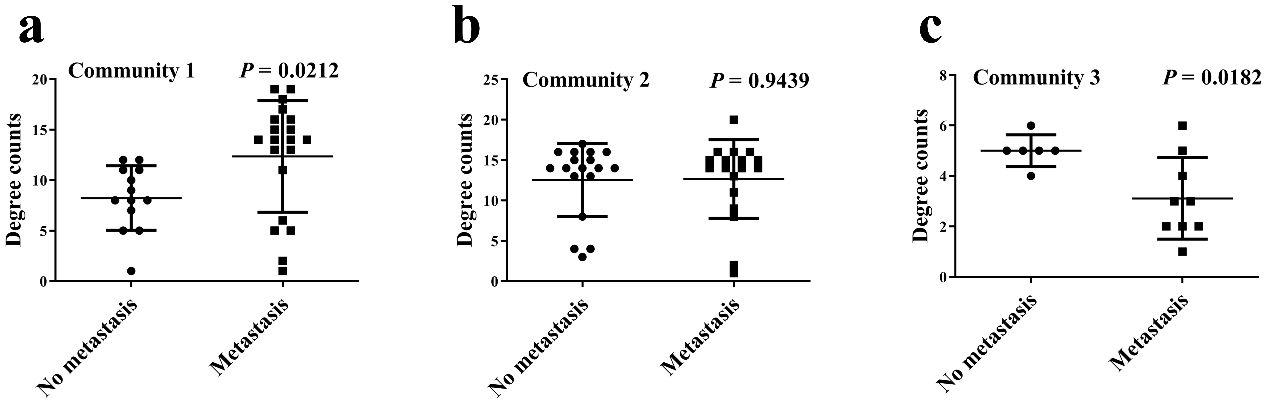


**Figure S8. Degree of lung mucosa-colonizing bacteria in metastatic and non-metastatic NSCLC patients.** Comparison of differences in degree in community 1 **(A)**, 2 **(B)**, and 3 **(C)** in metastatic (n = 29) and non-metastatic (n = 26) NSCLC patients. Confidence interval (CI) = 0.95.


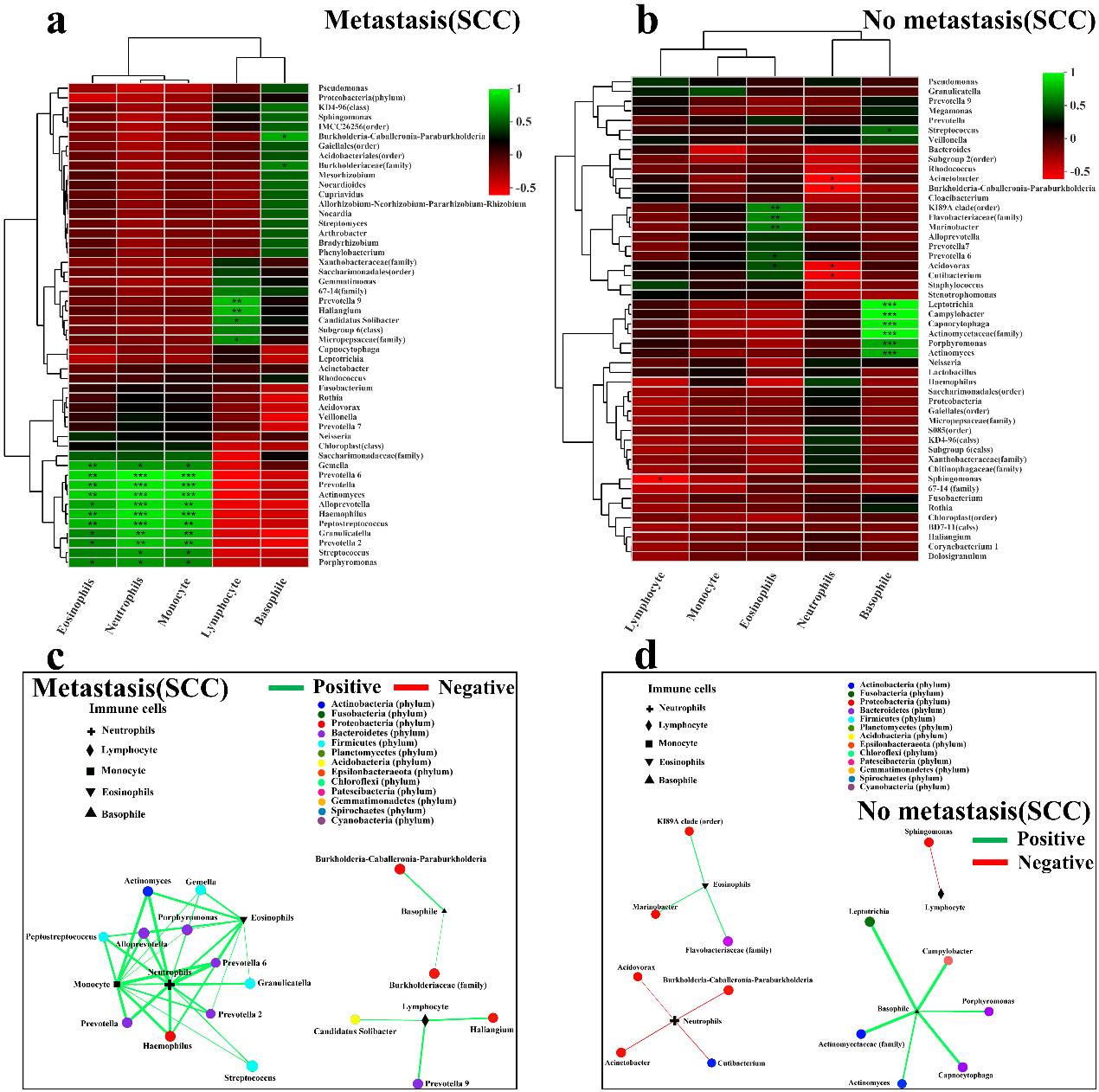


**Figure S9. Correlation between community members of lung mucosa-colonizing bacteria and immune cells in metastatic and non-metastatic SCC patients**. Correlation heatmap between lung mucosa-colonizing bacteria and immune cells (as environmental factors) in peripheral venous blood of metastatic (**A)** and non-metastatic (**B)** SCC patients. Correlations are positive (green) or negative (red) when R value is greater or less than 0, respectively. * 0.01 < *P* ≤ 0.05, ** 0.001 < *P* ≤ 0.01, *** *P* ≤ 0.001. Immune cells include neutrophils, monocytes, lymphocytes, eosinophils, and basophils. Two-way correlation network between lung mucosa-colonizing bacteria and immune cells (as environmental factors) in peripheral venous blood of metastatic (**C)** and non-metastatic (**D)** SCC patients. Correlation network was constructed based on Pearson rank correlation coefficients (∣Pearson Coef∣ ≥ 0.5, *P* < 0.05). Lines connecting different nodes indicate positive (green) or negative (red) correlation between bacteria and immune cells. For metastatic group and non-metastatic SCC patients n = 11 and 20, respectively. Correlation network and heatmap contain top 50 abundant bacteria. All bacteria were named to genus level unless noted otherwise in brackets.


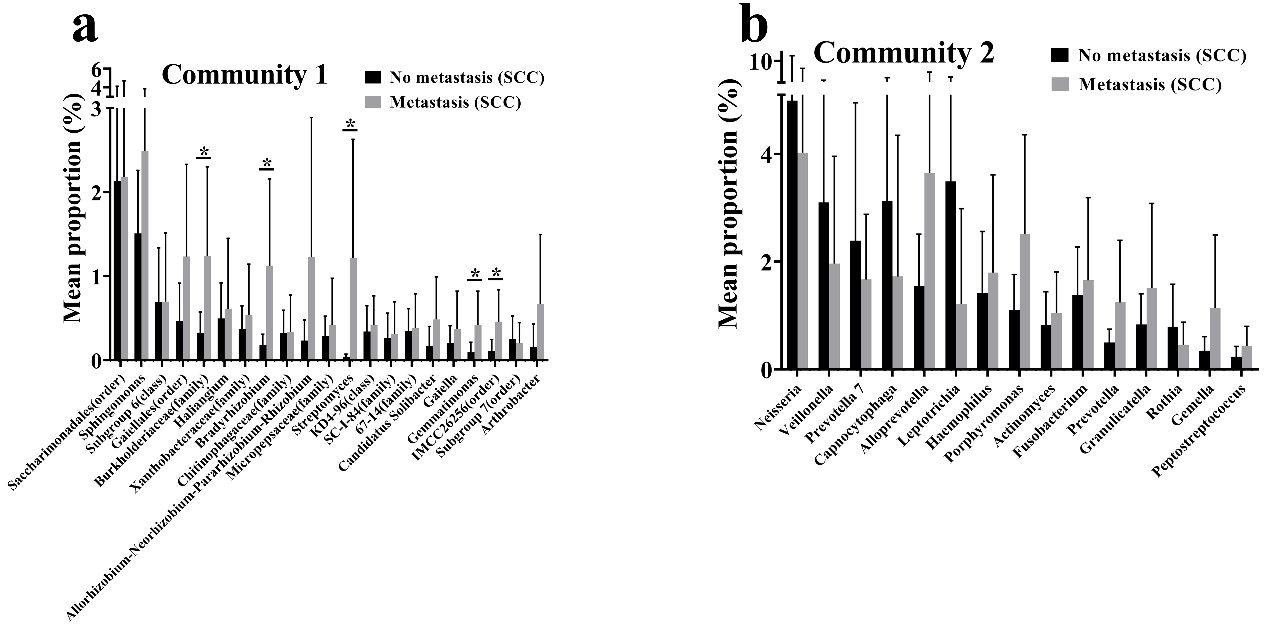


**Figure S10. Abundance comparison of community bacteria between metastatic and non-metastatic SCC patients.** Y axis represents relative bacterial abundance percentage (mean proportion) in community 1 (**A**) and 2(**B**) in metastatic (n = 11) and non-metastatic (n = 20) SCC patients. * 0.01 < *P* ≤ 0.05, confidence interval (CI) = 0.95. Comparison of bacterial abundance of top 50 abundant bacteria. All bacteria were named to genus level unless noted otherwise in brackets.


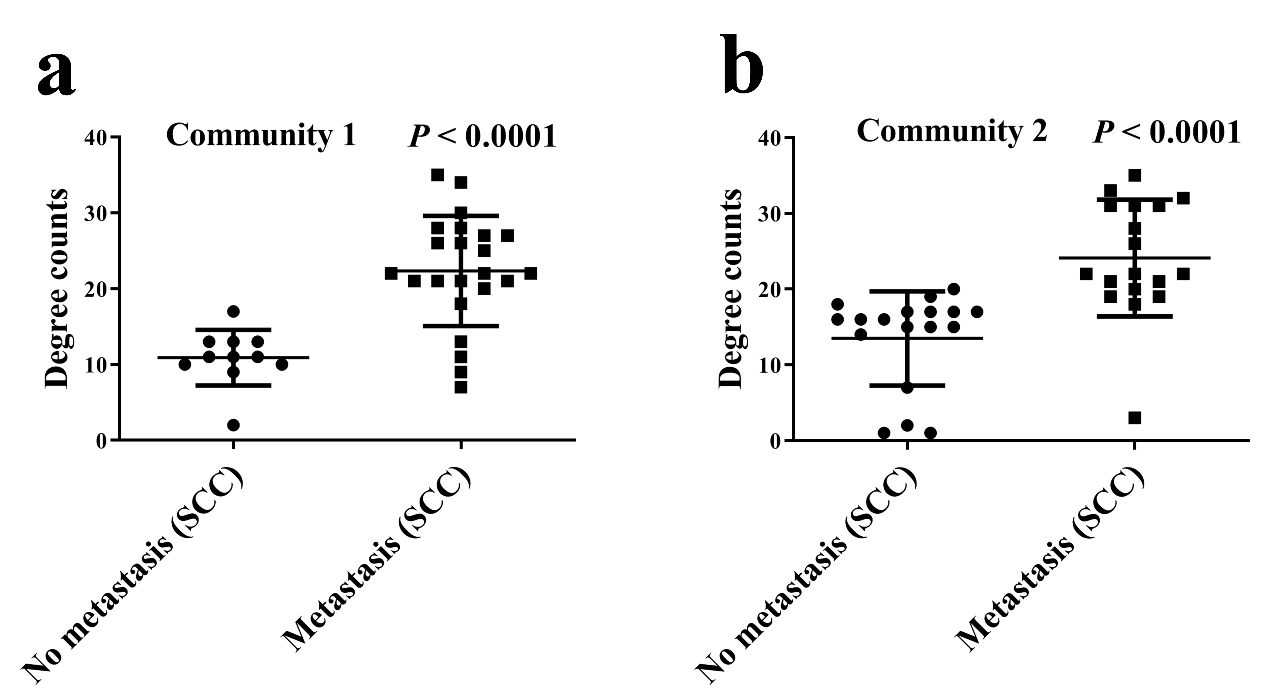


**Figure S11. Degree of lung mucosa-colonizing bacteria in metastatic and non-metastatic SCC patients.** Comparison of differences in degree in community 1 **(A)** and 2 **(B)** of metastatic (n = 11) and non-metastatic (n = 20) SCC patients. Confidence interval (CI) = 0.95.


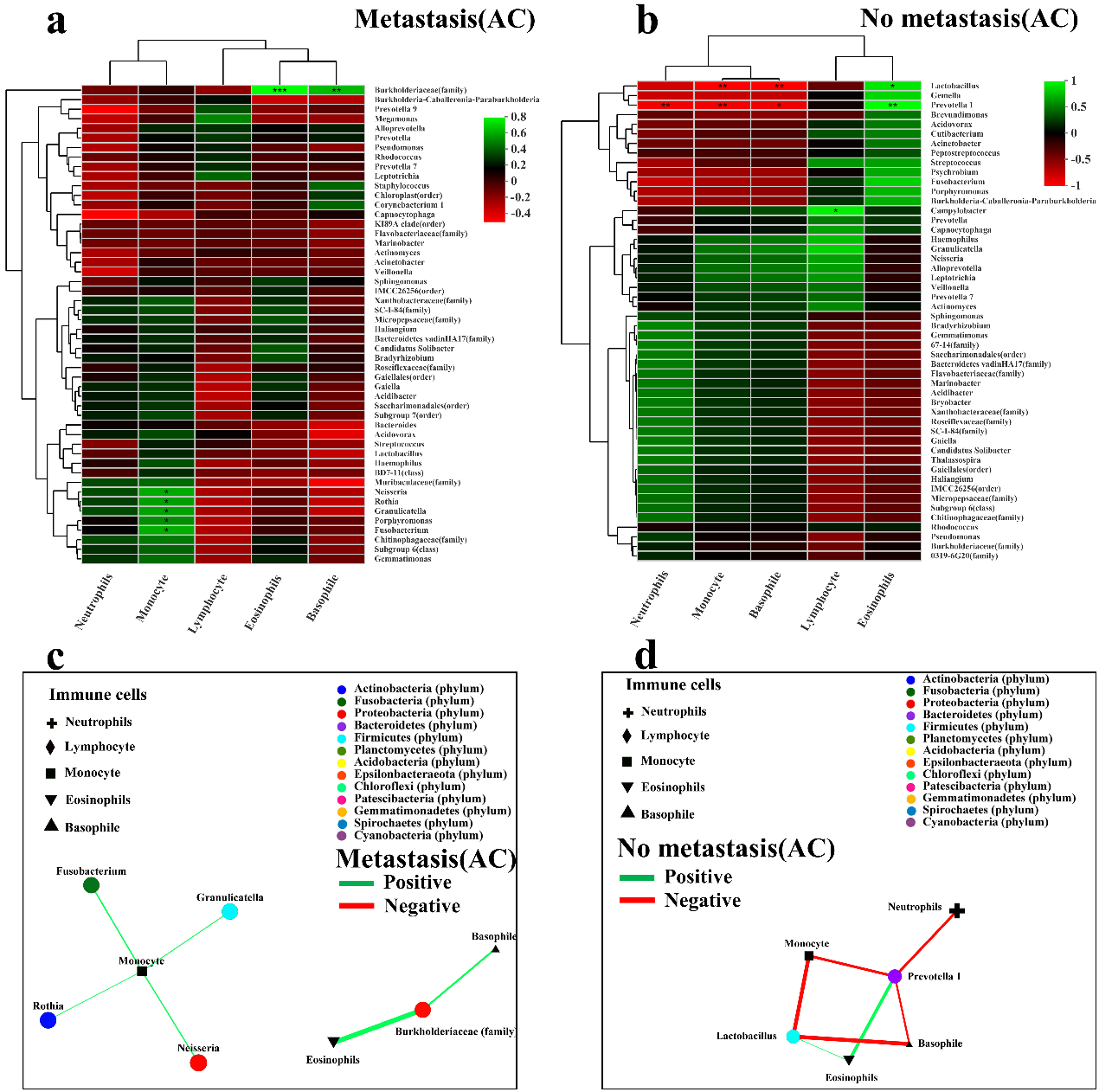


**Figure S12. Correlation between community members of lung mucosa-colonizing bacteria and immune cells in metastatic and non-metastatic AC patients**. Correlation heatmap between lung mucosa-colonizing bacteria and immune cells (as environmental factors) in peripheral venous blood of metastatic (**A)** and non-metastatic (**B)** AC patients. Correlations are positive (green) or negative (red) when R value is greater or less than 0, respectively. * 0.01 < *P* ≤ 0.05, ** 0.001 < *P* ≤ 0.01, *** *P* ≤ 0.001. Immune cells include neutrophils, monocytes, lymphocytes, eosinophils, and basophils. Two-way correlation network between lung mucosa-colonizing bacteria and immune cells (as environmental factors) in peripheral venous blood of metastatic (**C)** and non-metastatic (**D)** AC patients. Correlation network was constructed based on Pearson rank correlation coefficients (∣Pearson Coef∣ ≥ 0.5, *P* < 0.05). Lines connecting different nodes indicate positive (green) or negative (red) correlation between bacteria and immune cells. For metastatic and non-metastatic AC patients n = 18 and 6, respectively. Correlation network and heatmap contain top 50 abundant bacteria. All bacteria were named to genus level unless noted otherwise in brackets.


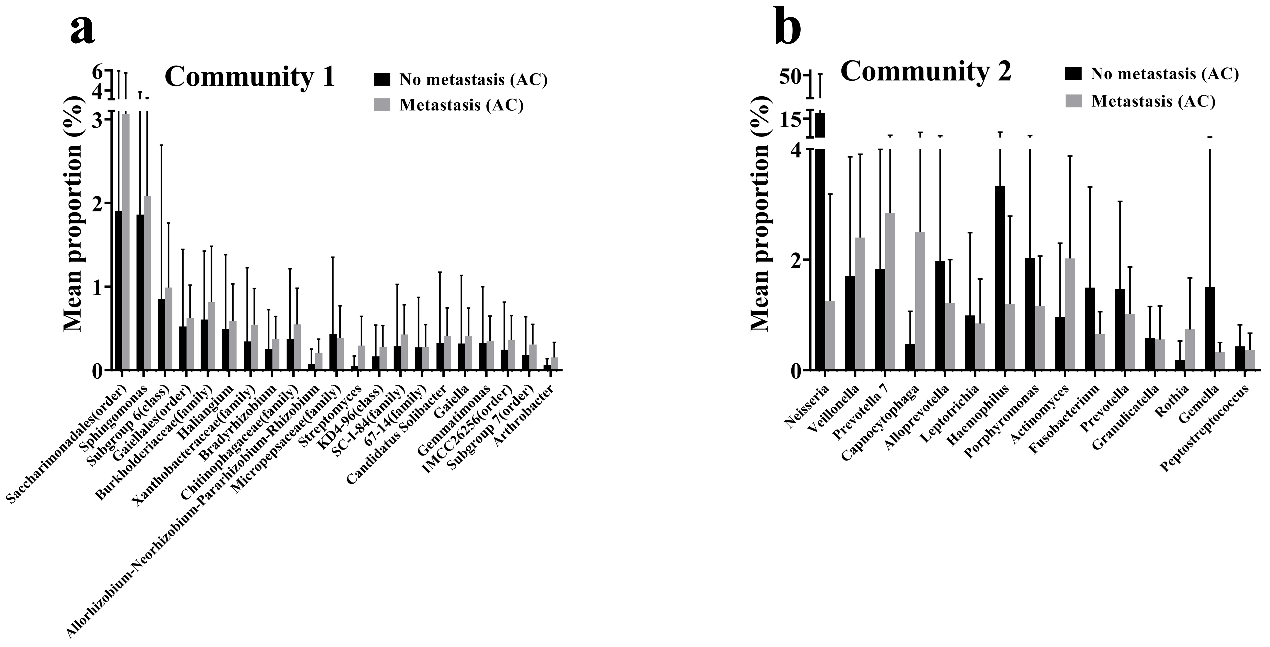


**Figure S13. Abundance comparison of community bacteria between metastatic and non-metastatic AC patients.** Y axis represents relative bacterial abundance percentage (mean proportion) in community 1 (**A**) and 2 (**B**) in metastatic (n = 18) and non-metastatic (n = 6) AC patients. Confidence interval (CI) = 0.95. Comparison of bacterial abundance of top 50 abundant bacteria. All bacteria were named to genus level unless noted otherwise in brackets.


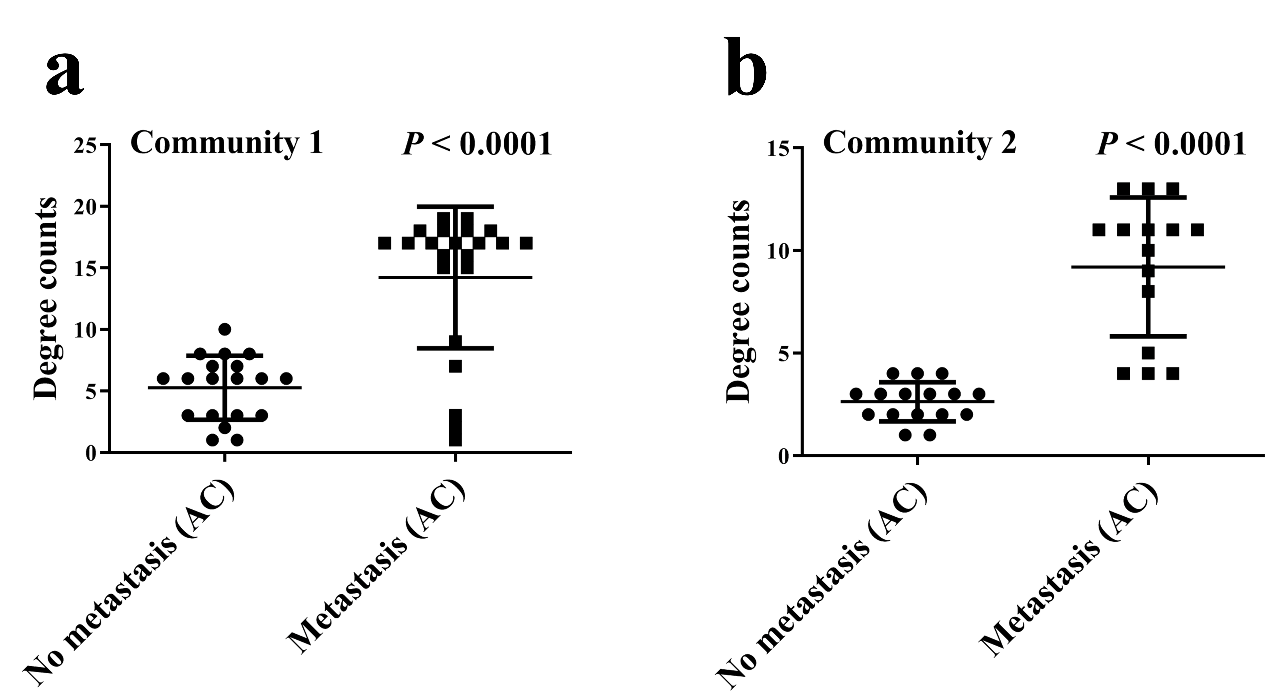


**Figure S14. Community degree of lung mucosa-colonizing bacteria in metastatic and non-metastatic AC patients.** Comparison of difference in degree in community 1 **(A)** and 2 **(B)** of metastatic (n = 20) and non-metastatic (n = 6) AC patients. Confidence interval (CI) = 0.95.


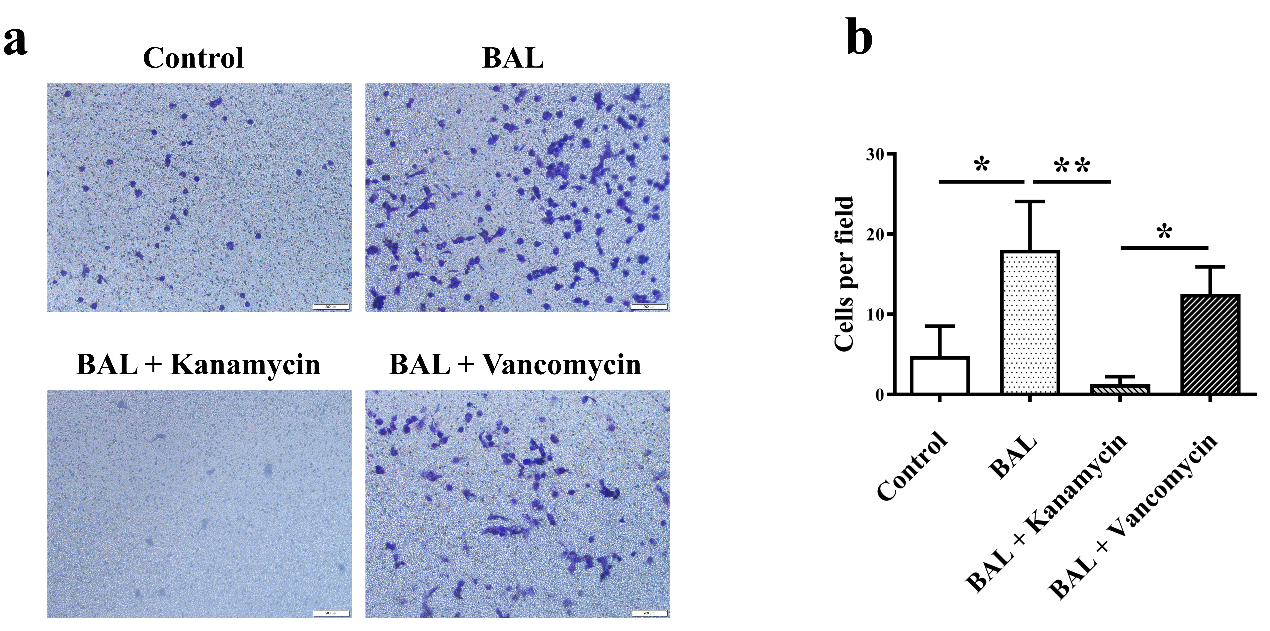


**Figure S15. Effects of bronchoalveolar lavage fluid from AC patients on NSCLC cell migration.** H1299 human NSCLC cell line with high metastasis potential was seeded into a 24-well transwell plate. At 24 h after seeding, bronchoalveolar lavage (BAL) fluid (50 μL) from AC patient 2# was added to the cell culture well. Vancomycin (2.5 μg/L) and kanamycin (5 μg/L) were also added for BAL + vancomycin group and BAL + kanamycin group, respectively. At 48 h after BAL and antibiotic management, migrated cells were stained (A) and counted (B).


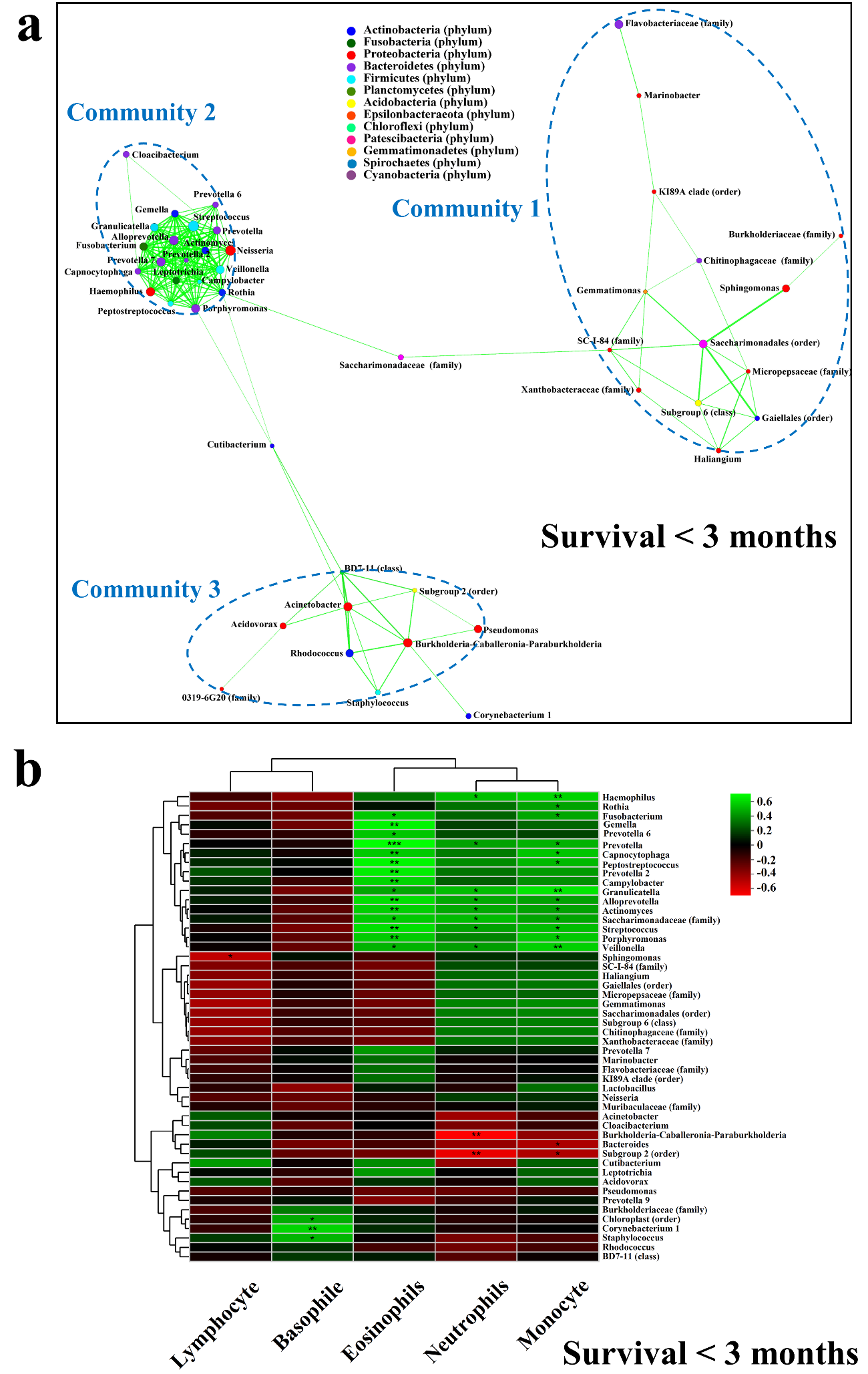


**Figure S16. Community structure of lung mucosa-colonizing bacteria (A), and the correlation between community members and immune cells (B) in NSCLC patients (n=18) with survivals of less than 3 months.** Correlation networks were constructed based on Spearman rank correlation coefficients (∣Spearman Coef∣ ≥ 0.5, *P* < 0.05). Dotted lines encircle different bacterial communities containing relatively stable inner members that are positively correlated with each other. Node diameter is positively related to bacterial abundance. Node colors indicate different bacterial phyla. Lines connecting different nodes indicate positive (green) or negative (red) correlation between bacteria, and line diameter is positively related to correlation value. Correlation network and heatmap contain top 50 abundant bacteria. All bacteria were named to genus level unless noted otherwise in brackets. Correlation heatmap between lung mucosa-colonizing bacteria and immune cells (as environmental factors) in peripheral venous blood of NSCLC patients. Correlations are positive (green) or negative (red) when R value is greater or less than 0, respectively. * 0.01 < *P* ≤ 0.05, ** 0.001 < *P* ≤ 0.01, *** *P* ≤ 0.001.


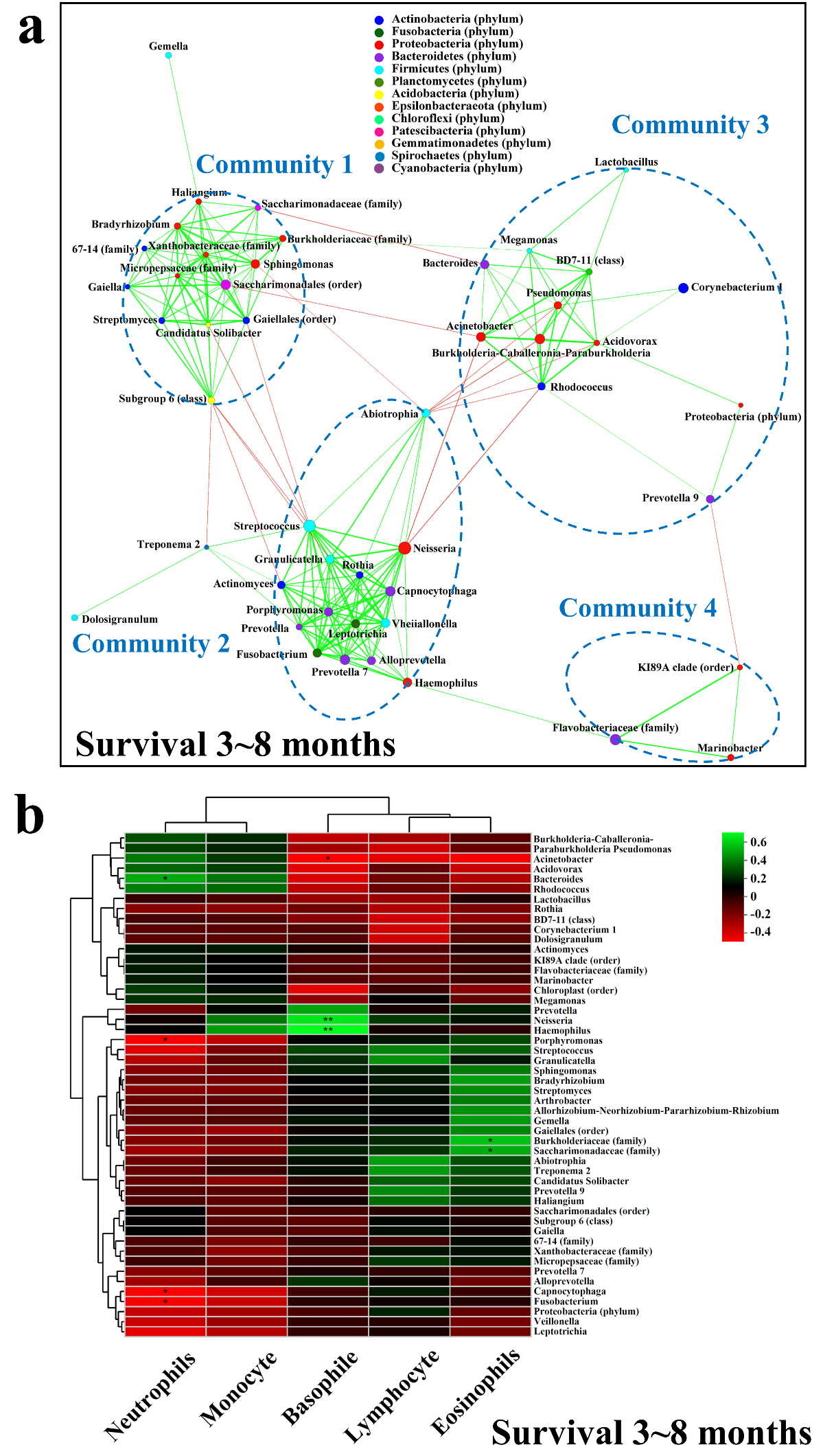


**Figure S17. Community structure of lung mucosa-colonizing bacteria (A), and the correlation between community members and immune cells (B) in NSCLC patients (n=24) with survivals of 3~8 months.** Correlation networks were constructed based on Spearman rank correlation coefficients (∣Spearman Coef∣ ≥ 0.5, *P* < 0.05). Dotted lines encircle different bacterial communities containing relatively stable inner members that are positively correlated with each other. Node diameter is positively related to bacterial abundance. Node colors indicate different bacterial phyla. Lines connecting different nodes indicate positive (green) or negative (red) correlation between bacteria, and line diameter is positively related to correlation value. Correlation network and heatmap contain top 50 abundant bacteria. All bacteria were named to genus level unless noted otherwise in brackets. Correlation heatmap between lung mucosa-colonizing bacteria and immune cells (as environmental factors) in peripheral venous blood of NSCLC patients. Correlations are positive (green) or negative (red) when R value is greater or less than 0, respectively. * 0.01 < *P* ≤ 0.05, ** 0.001 < *P* ≤ 0.01, *** *P* ≤ 0.001.


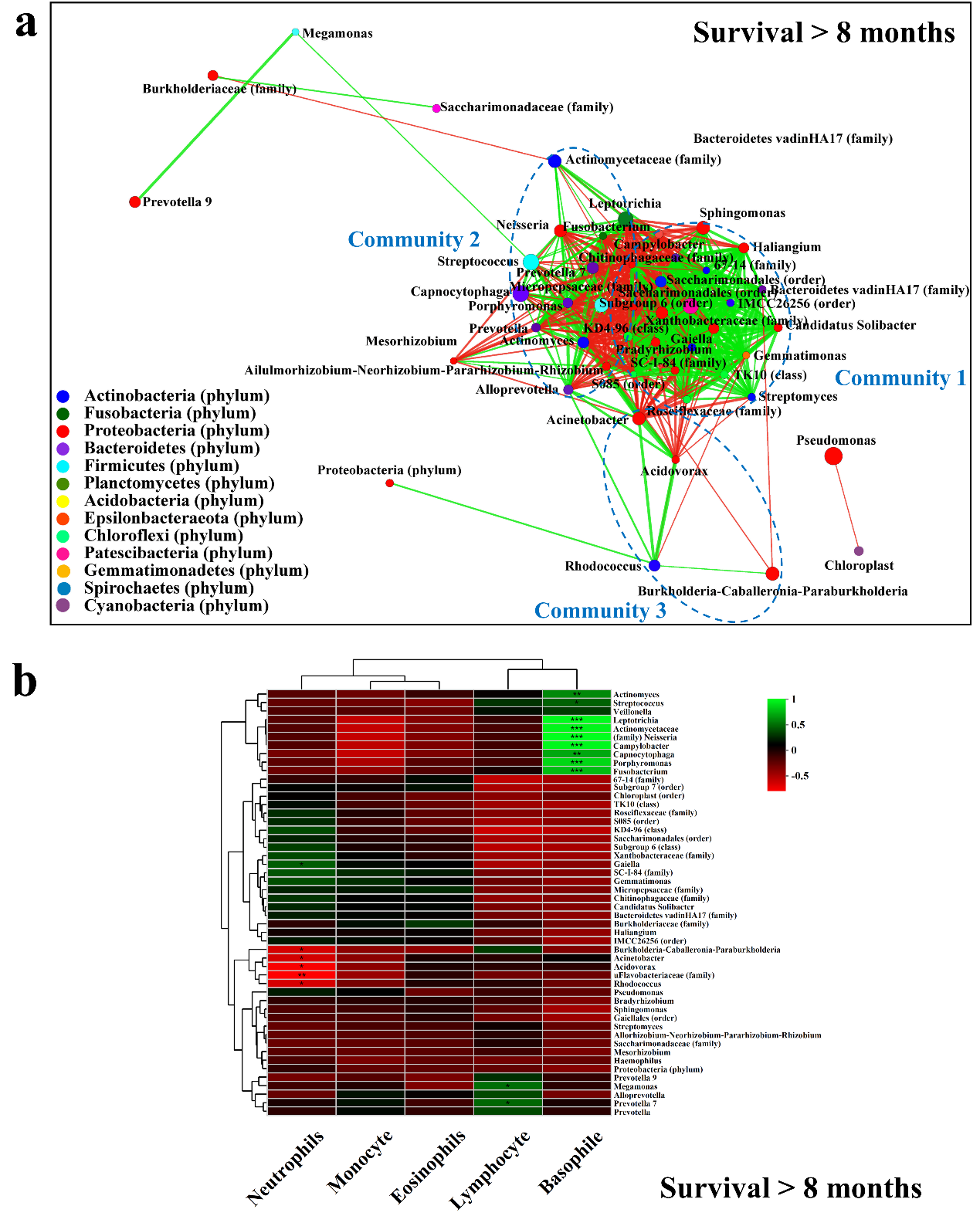


**Figure S18. Community structure of lung mucosa-colonizing bacteria (A), and the correlation between community members and immune cells (B) in NSCLC patients (n=15) with survivals of over 8 months.** Correlation networks were constructed based on Spearman rank correlation coefficients (∣Spearman Coef∣ ≥ 0.5, *P* < 0.05). Dotted lines encircle different bacterial communities containing relatively stable inner members that are positively correlated with each other. Node diameter is positively related to bacterial abundance. Node colors indicate different bacterial phyla. Lines connecting different nodes indicate positive (green) or negative (red) correlation between bacteria, and line diameter is positively related to correlation value. Correlation network and heatmap contain top 50 abundant bacteria. All bacteria were named to genus level unless noted otherwise in brackets. Correlation heatmap between lung mucosa-colonizing bacteria and immune cells (as environmental factors) in peripheral venous blood of NSCLC patients. Correlations are positive (green) or negative (red) when R value is greater or less than 0, respectively. * 0.01 < *P* ≤ 0.05, ** 0.001 < *P* ≤ 0.01, *** *P* ≤ 0.001.

**Supplementary Table**

**Table S1** Sample sequencing information statistics.

| **Groups** | **Samples** | **Seq number** | **Base number** | **Mean length** | **Mini length** | **Max length** |
| --- | --- | --- | --- | --- | --- | --- |
| **Healthy population** | H1 | 71122 | 30387372 | 427.256995 | 224 | 508 |
|  | H2 | 66963 | 28344324 | 423.2833654 | 220 | 497 |
|  | H3 | 70038 | 28943245 | 413.2505925 | 265 | 478 |
|  | H4 | 62329 | 26047446 | 417.9025173 | 213 | 499 |
|  | H5 | 74924 | 31115314 | 415.2916822 | 214 | 530 |
|  | H6 | 74323 | 30922722 | 416.0585821 | 201 | 503 |
|  | H7 | 61898 | 25519720 | 412.2866652 | 250 | 458 |
|  | H8 | 30283 | 12828177 | 423.6098471 | 200 | 509 |
|  | H9 | 30004 | 12568475 | 418.8933142 | 200 | 518 |
|  | H10 | 36566 | 15232356 | 416.5715692 | 208 | 529 |
|  | H11 | 39409 | 16649259 | 422.4735213 | 224 | 503 |
|  | H12 | 34567 | 14638607 | 423.4850291 | 211 | 513 |
|  | H13 | 29301 | 12364915 | 421.9963482 | 244 | 490 |
|  | H14 | 71537 | 29815944 | 416.790528 | 200 | 526 |
|  | H15 | 21140 | 8722885 | 412.6246452 | 207 | 507 |
|  | H16 | 53461 | 21691127 | 405.7373974 | 200 | 530 |
|  | H17 | 73600 | 30627378 | 416.1328533 | 252 | 464 |
|  | H18 | 29958 | 12598690 | 420.5450965 | 256 | 517 |
|  | H19 | 55552 | 23349170 | 420.31196 | 250 | 518 |
|  | H20 | 68856 | 28870777 | 419.2921024 | 208 | 502 |
|  | H21 | 24828 | 9926543 | 399.8124295 | 200 | 527 |
|  | H22 | 72437 | 30149303 | 416.2141309 | 204 | 524 |
|  | H23 | 51661 | 21856153 | 423.0687172 | 213 | 516 |
|  | H24 | 71883 | 30385132 | 422.702614 | 200 | 501 |
|  | H25 | 73814 | 30720450 | 416.1873086 | 210 | 495 |
|  | H26 | 21211 | 8793296 | 414.5630098 | 205 | 514 |
|  | H27 | 25393 | 10635182 | 418.8233765 | 248 | 505 |
|  | H28 | 27384 | 11557516 | 422.0536079 | 231 | 512 |
|  | H29 | 73829 | 31296130 | 423.9002289 | 214 | 482 |
|  | H30 | 65788 | 27790567 | 422.4260807 | 219 | 518 |
|  | H31 | 73741 | 31332823 | 424.9036899 | 259 | 497 |
|  | H32 | 22638 | 8764065 | 387.1395441 | 201 | 534 |
|  | H33 | 63790 | 26700310 | 418.5657627 | 219 | 471 |
|  | H34 | 70699 | 29146014 | 412.2549682 | 245 | 458 |
|  | H35 | 38589 | 16189660 | 419.5408018 | 250 | 464 |
|  | H36 | 69119 | 28798823 | 416.6556663 | 231 | 486 |
|  | H37 | 31825 | 13388634 | 420.695491 | 249 | 507 |
|  | H38 | 52450 | 22153342 | 422.3706768 | 229 | 531 |
|  | H39 | 56127 | 23533876 | 419.2968803 | 200 | 518 |
|  | H40 | 59379 | 24762469 | 417.0240152 | 232 | 519 |
|  | H41 | 70301 | 29258154 | 416.1840372 | 202 | 505 |
|  | H42 | 41726 | 17512852 | 419.7107798 | 211 | 517 |
|  | H43 | 65815 | 27449577 | 417.0717466 | 202 | 518 |
|  | H44 | 37678 | 15597951 | 413.9803334 | 204 | 509 |
|  | H45 | 70639 | 29846689 | 422.5242288 | 241 | 505 |
|  | H46 | 27335 | 11476531 | 419.8474849 | 210 | 519 |
|  | H47 | 40383 | 17029571 | 421.7014833 | 215 | 525 |
|  | H48 | 70083 | 28865507 | 411.8760184 | 345 | 482 |
|  | H49 | 50218 | 21213084 | 422.4199291 | 224 | 529 |
|  | H50 | 66348 | 27504187 | 414.5443269 | 214 | 458 |
| **SCC patients** | SCC1 | 50834 | 21629306 | 425.4889641 | 220 | 506 |
|  | SCC2 | 48981 | 20013662 | 408.6005186 | 200 | 526 |
|  | SCC3 | 38965 | 16184461 | 415.3589375 | 207 | 510 |
|  | SCC4 | 44093 | 18431189 | 418.007144 | 203 | 521 |
|  | SCC5 | 63671 | 27017293 | 424.3265066 | 201 | 492 |
|  | SCC6 | 22805 | 9578572 | 420.0206972 | 207 | 431 |
|  | SCC7 | 47940 | 20353180 | 424.5552774 | 252 | 447 |
|  | SCC8 | 74146 | 31097186 | 419.404769 | 200 | 539 |
|  | SCC9 | 74930 | 31433362 | 419.5030295 | 222 | 534 |
|  | SCC10 | 37256 | 15478258 | 415.4567855 | 200 | 514 |
|  | SCC11 | 73537 | 31175863 | 423.9479854 | 218 | 521 |
|  | SCC12 | 63913 | 26978213 | 422.1083817 | 207 | 505 |
|  | SCC13 | 52766 | 21167470 | 401.1573741 | 200 | 512 |
|  | SCC14 | 29562 | 12406319 | 419.6711657 | 250 | 526 |
|  | SCC15 | 56067 | 23762448 | 423.8223554 | 201 | 514 |
|  | SCC16 | 21233 | 8873236 | 417.8983658 | 200 | 528 |
|  | SCC17 | 72708 | 30083929 | 413.7636711 | 259 | 498 |
|  | SCC18 | 70576 | 29593657 | 419.3161556 | 253 | 502 |
|  | SCC19 | 70487 | 29855423 | 423.5592804 | 253 | 454 |
|  | SCC20 | 51482 | 21655586 | 420.6438367 | 207 | 517 |
|  | SCC21 | 36850 | 15381852 | 417.4179647 | 201 | 532 |
|  | SCC22 | 74194 | 30761263 | 414.6058037 | 231 | 464 |
|  | SCC23 | 21207 | 8894037 | 419.3915688 | 243 | 518 |
|  | SCC24 | 48702 | 20214444 | 415.0639399 | 239 | 448 |
|  | SCC25 | 42990 | 17813280 | 414.3586881 | 200 | 517 |
|  | SCC26 | 28593 | 12088039 | 422.7621796 | 233 | 515 |
|  | SCC27 | 73397 | 31119445 | 423.9879695 | 214 | 497 |
|  | SCC28 | 72006 | 29746449 | 413.1106991 | 223 | 501 |
|  | SCC29 | 60261 | 24982744 | 414.5756625 | 206 | 522 |
|  | SCC30 | 74876 | 31200228 | 416.6919707 | 226 | 517 |
|  | SCC31 | 52705 | 21515081 | 408.2170762 | 207 | 531 |
|  | SCC32 | 28229 | 11944475 | 423.1278118 | 214 | 531 |
|  | SCC33 | 41729 | 17499794 | 419.3676819 | 207 | 518 |
| **AC patients** | AC1 | 74092 | 30858429 | 416.4880014 | 220 | 523 |
|  | AC2 | 30644 | 12533708 | 409.0101814 | 203 | 519 |
|  | AC3 | 69404 | 29492700 | 424.9423664 | 247 | 496 |
|  | AC4 | 68032 | 28426730 | 417.8435148 | 200 | 531 |
|  | AC5 | 41767 | 17314857 | 414.5583116 | 201 | 534 |
|  | AC6 | 50924 | 21140644 | 415.141073 | 200 | 537 |
|  | AC7 | 16181 | 6704519 | 414.3451579 | 237 | 505 |
|  | AC8 | 37262 | 15702130 | 421.3979389 | 204 | 519 |
|  | AC9 | 41611 | 17602687 | 423.0296556 | 220 | 492 |
|  | AC10 | 47617 | 19919447 | 418.326375 | 202 | 532 |
|  | AC11 | 71355 | 29968406 | 419.990274 | 231 | 520 |
|  | AC12 | 60163 | 25401380 | 422.2093313 | 225 | 508 |
|  | AC13 | 55970 | 23200957 | 414.5248705 | 203 | 485 |
|  | AC14 | 49124 | 20251377 | 412.2501629 | 201 | 534 |
|  | AC15 | 67216 | 27924979 | 415.4513657 | 214 | 466 |
|  | AC16 | 69762 | 29879375 | 428.3044494 | 213 | 503 |
|  | AC17 | 37515 | 15629084 | 416.6089298 | 200 | 518 |
|  | AC18 | 72030 | 30397024 | 422.0050535 | 210 | 523 |
|  | AC19 | 35810 | 15031304 | 419.7515778 | 206 | 492 |
|  | AC20 | 25673 | 10804429 | 420.8479336 | 224 | 539 |
|  | AC21 | 50787 | 21298226 | 419.3637348 | 201 | 505 |
|  | AC22 | 38665 | 16242503 | 420.0828398 | 231 | 534 |
|  | AC23 | 42698 | 18104263 | 424.0072837 | 207 | 525 |
|  | AC24 | 47653 | 20000393 | 419.7090005 | 200 | 508 |
